# Financial inclusion and stability in Ethiopia using bank-level data: A two-step system GMM estimation

**DOI:** 10.12688/f1000research.158461.1

**Published:** 2024-11-15

**Authors:** Mohammed Arebo, Filmon Hando, Andualem Mekonnen

**Affiliations:** 1 Regional and Local Development Studies, Addis Ababa University College of Development Studies, Addis Ababa, Ethiopia

**Keywords:** Financial Inclusion; Principal Component Analysis; Bank stability; GMM; Ethiopia

## Abstract

**Background:**

This paper examines the impact of financial inclusion on bank stability within Ethiopian context, using panel data from 17 commercial banks over the period 2015-2023. Given the scarcity of research focused on the relationship between financial inclusion and bank stability in Ethiopia, this paper seeks to address a crucial gap by analyzing both conventional and digital aspects of financial inclusion in relation with bank stability.

**Methods:**

A two-stage principal component analysis (PCA) was conducted to construct a composite financial inclusion index, integrating 10 conventional and 5 digital indicators. The study applied a two-step robust system generalized method of moments (GMM) to examine the effects of financial inclusion on bank stability, complemented by Granger causality testing to examine the directionality of this relationship.

**Results:**

The result reveals a significant positive effect of financial inclusion on bank stability and Granger causality tests confirms a bi-directional relationship between financial inclusion and stability, indicating that improvements in financial inclusion foster greater stability and vice versa. Our results also highlight that while bank efficiency and GDP growth rate positively effect stability, total assets and income diversification exhibit detrimental effects.

**Conclusions:**

It is essential to capitalize policy synergies to promote bank stability and to enhance financial inclusion through conventional and digital channels, while carefully managing the implications of risks associated with income diversification and asset distribution. Ensuring inclusive financial system is vital for maintaining bank stability, thus positioning it as a key priority for financial institutions.

## 1. Introduction

In contemporary financial policymaking, the pursuit of financial system stability has increasingly been viewed in alongside with the promotion of financial inclusion (FI) ( Antwi et al., 2024; Arora, 2020). Growing evidence suggests that the degree of FI can siginificantly impact the stability of financial systems ( Damane & Ho, 2024; Antwi et al., 2024; Vo et al., 2020; Kim et al., 2020; Ahamed & Mallick, 2019). Historically, the dominant focus of financial authorities was primarily on preserving financial stability ( Schinasi, 2005). However, over recent decades, FI has risen to prominence as a key policy goal, complementing the objectives of stability, integrity, and consumer protection, largely driven by the initiatives of global regulatory bodies and stakeholders ( Hannig & Jansen, 2010). This shift prompts a critical inquiry into whether financial stability and FI function as substitutes or complements within the broader regulatory framework ( Morgan & Pontines, 2017).

This research investigates the impact of FI on bank stability, focusing on Ethiopia to address existing literature gaps. Bank stability, characterized by the absence of crises within the sector, is essential for maintaining public confidence and ensuring the effective execution of financial intermediation tasks ( Schinasi, 2004). The determinants of bank stability can differ significantly across various contexts, requiring tailored policy responses ( Ozili, 2018). Factors influencing bank stability include internal elements such as general ledger (GL) and profit-loss (PL) accounts, as well as external socio-economic and political conditions ( Ha & Nguyen, 2023). FI, which encompasses improvements in accessibility, quality, quantity, and efficiency of financial services ( Pesqué-Cela et al., 2021; Demirgüç-Kunt et al., 2017; Allen et al., 2016; Babajide et al., 2015), has been demonstrated to enhance livelihoods, create opportunities, and bolster economies ( Le et al., 2019).

Empirical studies on the impact of FI on bank stability in developing countries is scarce ( Chinoda & Kapingura, 2023; Koudalo & Toure, 2023; Jungo et al., 2022). Neglecting this relationship could lead to severe consequences, including costly financial crises or ongoing financial exclusion ( Danisman & Tarazi, 2020; Čihák et al., 2016). Schoenmaker (1996) highlights that failures within individual financial sectors can trigger contagion effects, leading to widespread instability stemming from the collapse of financial institutions. In such scenarios, a single institution's failure can create a ripple effect, eroding investor confidence and prompting a rush to liquidate deposits ( Huynh et al., 2020). Therefore, understanding contagion risk is crucial for comprehending the dynamics of the financial system and its stability ( Huynh et al., 2020). Consequently, policymakers must adeptly balance the trade-offs and synergies between fostering FI and maintaining stability ( Damane & Ho, 2024; Čihák et al., 2016).

In Ethiopia, while several studies have been conducted in relation to financial sector stability ( Filatie & Sharma, 2024; Kebede et al., 2024; Fisseha, 2023; Yitayaw et al., 2023; Dress, 2022; Abdu, 2021; Isayas & McMillan, 2021; Aman, 2019; Meher & Getaneh, 2019; Zelie & Wassie, 2019; Meressa, 2018), the impact of FI on bank stability remains largely unexplored. Additionally, recent data underscore significant disparities in financial service provision, with loan disbursement heavily concentrated in urban areas (99.8%) and a troubling concentration of loans among the top ten borrowers, who hold 23.5% of all outstanding loans ( NBE Financial Stability Report, 2024), highlight the need for a thorough examination of how FI effect bank stability.

To the best of the researchers' knowledge, no prior study has examined the effect of FI on bank stability in Ethiopia. This study makes several contributions. First, it constructs a composite FI index using two-stage PCA, incorporating both conventional and digital dimensions. Second, it applies the two-step robust system GMM estimator to examine the impact of FI on bank stability while addressing endogeneity concerns. Third, it employs panel causality tests, including those by Juodis et al. (2021) and Dumitrescu and Hurlin (2012), to determine the causal direction between FI and stability. Finally, the study offers actionable insights for policymakers, guiding strategies to enhance both FI and financial stability in Ethiopia.

The remainder of the study is structured as: Part 2 reviews the literature; Part 3 details the data sources and econometric methods; Part 4 presents and discusses the empirical results; and Part 5 concludes with key findings and policy implications.

## 2. Literature review

### 2.1 Theoretical literature review

FI, from a macroeconomic perspective, is seen as a catalyst for “creative destruction,” promoting economic growth, reducing inequality, and enhancing financial stability. However, at the micro level, excessive FI can lead to opportunistic behaviors, such as fund misuse and deliberate payment delays, which may undermine financial stability ( Hua et al., 2023). The impacts of FI are neither uniform nor static; they vary across different temporal and spatial contexts. This has led to divergent views among researchers regarding its impact on bank stability, with two divergent schools of thought emerging as the one positing a FI-stability effect and the other suggesting a FI-instability outcome ( Damrah et al., 2023; Moaz, 2022). To that end, the relationship between FI and bank stability has become a central theme in both academic and policy discourse ( Čihák et al., 2016). This study employs a multi-theoretical approach, drawing on asymmetric information theory, financial intermediation theory, systemic risk theory, and portfolio theory. These theoretical underpinnings collectively offer a nuanced understanding of the pathways through which FI can effect bank stability in Ethiopia.

Asymmetric information theory, highlighted by Akerlof (1970), addresses the challenges posed by uneven information distribution in financial markets. In FI, this imbalance is particularly significant as institutions serve previously unbanked populations. Difficulties in assessing creditworthiness can lead to adverse selection, hindering credit risk management and operational efficiency, which may compromise financial stability ( Oanh et al., 2023; Moaz, 2022; Sethy & Goyari, 2022; Barik & Pradhan, 2021; Khan, 2011; Bofondi & Gobbi, 2003). Balancing information asymmetry within FI context can thus strengthen the financial system.

The financial intermediation theory underscores the pivotal role of banks in linking savers with borrowers ( Scholtens & Wensveen, 2003; Diamond, 1984), thereby facilitating FI. By providing liquidity, managing risks, and bridging informational gaps, banks contribute to the efficient allocation of capital and the mitigation of risks, which enhances overall financial stability ( Servigny & Renault, 2004). This theory emphasizes that by expanding financial access, particularly in developing economies where financial exclusion is significant, banks can improve their performance by broadening their customer base, increasing deposits, and fostering a more resilient financial system ( Kim et al., 2018; Diamond, 1984). This phenomenon lessens information asymmetry, thereby lessens market imperfections ( Oanh et al., 2023).

Systemic risk theory, as developed by Minsky (1982) and later refined by Borio (2011), addresses the interconnectedness of financial institutions and the amplification of shocks through mechanisms such as leverage, asset price bubbles, and procyclicality. Borio differentiates systemic risk into temporal and cross-sectional dimensions. Temporal risk involves the dynamic evolution of aggregate risk, while cross-sectional risk pertains to the distribution of risk within the financial system, influenced by common exposures and counterparty risks. Within the context of FI, Khan (2011) suggests that expanding borrower bases can lower lending standards, potentially destabilizing the financial system. Conversely, Hannig and Jansen (2010) argue that FI can enhance stability by diversifying deposit and loan bases, involving segments less susceptible to economic cycles, while Prasad (2010) highlights the positive effects of credit access on employment and economic growth.

Portfolio theory highlights the significance of diversification in mitigating risk ( Markowitz, 1952). In the context of FI, a diversified loan portfolio can potentially reduce the impact of credit losses on individual banks and the overall financial system ( Beck et al., 2013; Wang & Lin, 2021). Adem (2022) concurs, noting that diversification lowers bankruptcy risk for banks. Khan (2011) outlines three main ways of mitigating the FI on financialinstability: diversification of bank assets through increased lending to smaller firms reduces portfolio risk; an expanded and stable deposit base lessens reliance on volatile non-core financing; and improved monetary policy transmission enhances stability. However, as Huynh & Dang (2021) argue, the effectiveness of diversification hinges on the correlation between loan segments and the portfolio's overall risk profile.

Drawing on these theoretical foundations, this study offers a unique lens through which the impact of FIonbank stability can be comprehended. The theories collectively guide our understanding of how FI initiatives can be designed to support the broader financial servicesaccess and the long-term stability of the financial system.

### 2.2 Empirical review

Empirical research regarding the impact of FI on the stability of financial systems presents varied outcomes ( Damrah et al., 2023; Feghali et al., 2021; Vo et al., 2020). Despite data limitations, these studies provide perspectives on both the positive and negative effects of FI on the stability of banks.

**2.2.1 Positive impact of financial inclusion on bank stability**

Wang and Luo (2022), using data from over 1500 commercial banks across 36 emerging economies, found that FI significantly enhances bank stability by promoting operational efficiency, risk management, and funding stability. Similarly, Nguyen and Du (2022), employing System GMM for 102 banks in six ASEAN countries, concluded that FI promotes stability through increased customer deposits and reduced non-performing loans (NPLs). Their findings align with those of Morgan and Pontines (2017), who showed that lending to small and medium sized enterprises (SMEs) diversifies risk and contributes to higher Z-scores and lower NPL ratios, key indicators of bank stability. These studies collectively highlight how extending credit to SMEs serves as a buffer against financial instability by diversifying loan portfolios and reducing concentrated risk exposure.

Ahamed and Mallick (2019) extended this argument by analyzing a global dataset of 2635 banks in 86 countries. Using two-step system GMM, they posited that FI enhances stability by increasing customer deposits and lowering marginal banking costs, with the effectiveness of these benefits being contingent on institutional quality. They highlight the role of a robust institutional framework in ensuring that FI does not devolve into unchecked risk-taking. In this context, the study implicitly warns against viewing FI initiatives in a silo, devoid of regulatory oversight or institutional support. Similarly, Hannig and Jansen (2010) emphasized the role of robust institutional frameworks in ensuring that FI initiatives do not lead to unchecked risk-taking, a finding also supported by Sethy and Goyari (2022) in their study of South Asian countries, where FI was found to be a key driver of long-term stability.

Vo et al. (2020) and Chinoda and Kapingura (2023) both underscore the importance of FI in reducing systemic risks. Vo et al. (2020) highlight its role in broadening the depositor base and improving financial resilience in emerging markets, while Chinoda and Kapingura (2023) emphasize the stabilizing effects of digital FI in Sub-Saharan Africa, noting that it significantly reduces NPLs and enhances bank stability. Another layer of analysis emerges in the work of López and Winkler (2019), who explore FI during periods of financial crises using 189 economies data for the period of 2004–2017. Their study finds that countries with higher levels of FI experience less severe disruptions in credit availability during economic downturns. Similarly, Pham and Doan (2020), examining 42 countries across Asia using Feasible Generalized Least Squares (FGLS), conclude that even a weak positive impact of FI on stability is enough to safeguard financial markets against extreme volatility, especially when FI fosters a stable retail deposit base.

In the MENA region, Neaime and Gaysset (2018) demonstrated that FI contributes positively to stability by reducing income inequality. This finding is echoed by Malik et al. (2022), who showed that in Asian countries, FI mediates the relationship between governance quality and financial stability, further emphasizing its stabilizing potential when integrated with good governance practices. Similarly, Koudalo and Toure (2023), using Fixed Effect and GMM models for 54 African countries, found a positive association between account penetration, a key measure of FI, and stability, particularly under conditions of income equality, political stability, and financial openness.

Khan (2011) and Čihák et al. (2016) underscore the critical role of institutional quality and regulation in mediating the relationship between FI and stability. Khan (2011) identified three key channels: asset diversification, the inclusion of small depositors, and enhanced monetary policy transmission. Čihák et al. (2016) found that FI strengthens stability in countries with robust supervisory frameworks. These insights suggest that FI must be part of a comprehensive approach to financial stability, integrating regulatory quality, institutional capacity, and risk management. Dienillah et al. (2018), using a Tobit model for 19 countries from 2004 to 2014, revealed that FI positively impacts stability in high-income nations but shows insignificant effects in lower-income groups. This highlights the need for tailored FI strategies suited to specific economic and regulatory environments. Jungo et al. (2022) further emphasize the importance of expanding financial access and encouraging investment in FI to maintain monetary system stability, particularly in developing countries.

**2.2.2 Negative impact of financial inclusion on bank stability**

Damrah et al. (2023) present a nuanced view of FI’s impact on bank stability in Kuwait from 2003 to 2017, illustrating how inclusion can have both positive and negative effects depending on the type of bank (Islamic vs. conventional) and external factors such as financial crises. Their Linear Mixed Model (LMM) reveals that while FI improves access to services, it also introduces inefficiencies and increased risk exposure, particularly during times of economic distress. Their findings align with those of Barik and Pradhan (2021), who, using system-GMM estimators for BRICS nations from 2005 to 2015, argue that rapid credit expansion facilitated by FI can deteriorate lending standards and escalate non-performing loans (NPLs).

Employing System GMM ranging from 2011-2019, Umar and Akhtar (2024), examining Chinese banks, found that while FI generally reduces bank risk-taking, it can increase risks in unlisted and large banks, suggesting that the impact of FI varies depending on bank characteristics. Similarly, Le et al. (2019), using Feasible Generalized Least Squares (FGLS), highlight the trade-offs associated with FI, noting that while FI promotes financial sustainability, it also negatively impacts financial efficiency due to the operational challenges of servicing previously unbanked populations. Hua et al. (2023) extend this argument by proposing an inverted U-shaped relationship between FI and financial stability, demonstrating that beyond a certain threshold, further inclusion can destabilize the financial system, particularly in countries with weak regulatory environments.

Kebede et al. (2021) further emphasize the intricate nature of FI in Africa, noting that its benefits may be limited by high market concentration, which can stifle competition and reduce the effectiveness of inclusion efforts. This view is echoed by Oanh et al. (2023) and Jima and Makoni (2023), who highlight the contextual nature of FI’s impact. Employing panel vector autoregression (PVAR) model for 58 countries (27 low financial development states and 31 high financial development states) from 2004 to 2020, Oanh et al. (2023) found that FI positively correlates with stability in low-financial-development countries, but this relationship turns negative in more developed financial systems, where excessive credit access may heighten systemic risks. Similarly, Jima and Makoni (2023), using ARDL cointegration and Granger causality tests from 2000 to 2019 for 26 Sub-Saharan African countries, revealed that while FI contributes to both short-term and long-term stability, the relationship is bidirectional and contingent on broader economic and institutional conditions. In addition, Damane & Ho (2024) and Čihák et al. (2016), who explore the synergies and trade-offs between FI and stability. They argue that while FI enhances access to services, it does not necessarily lead to their optimal use, and the resultant credit expansion can introduce new risks if not managed appropriately. This perspective challenges the notion that FI should be pursued in isolation, instead advocating for a more integrated approach that considers the full spectrum of financial services and their impacts on both individuals and institutions.

The empirical literature on FI and financial stability offers a rich diverse of findings, suggesting that a recurring theme across both streams of research is the need to avoid viewing FI or stability in a silo. This concise review of the literature is crucial for our empirical analysis. Prior research seems to have predominantly overlooked the impact of FI on stability, particularly in the context of the Ethiopian banking sector. This study employs bank-level data to offer appropriately tailored policy implication aimed at enhancing bank stability and FI in Ethiopia.

## 3. Methods

### 3.1 Source and sample of data

Commercial banking institutions in Ethiopia are the most dominant and primary point of access to essential financial services. Based on the data compiled from NBE financial stability report (2024), commercial banks hold for 91.2% of total assets, 97.8% of deposits, 93.9% of credit, and 76.1% of equity in the financial sector, as of June 30, 2023. In light of this dominance, the study focuses on commercial banks but due to data availability concern and the newness of other banks, the researchers focuses on 17 commercial banks. Data for the study sourced from various reliable institutions, like National Bank of Ethiopia (NBE), Commercial banks (CBs) annual report, and World Bank (WB) covering the period 2015-2023.

### 3.2 Construction and identification of variables

**3.2.1 Dependent variables**

In this research, Z-score is used as a measure of bank stability, following established literature (e.g., Damrah et al., 2023; Koudalo & Toure, 2023; Barik & Pradhan, 2021; Sethy & Goyari, 2022; Vo et al., 2020; Ahamed & Mallick, 2019). Z-score is valued for its ability to indicate bank insolvency risk, reflecting the likelihood that a bank's assets may not cover its liabilities ( Koudalo & Toure, 2023). Higher returns on assets and greater capitalization improve stability, while lower figures suggest increased risk ( Damrah et al., 2023). This score, calculated from return on assets, volatility, and leverage ( Damrah et al., 2023; Vo et al., 2020), is constructed in this study as follows:

$$Z-{\text{score}}_{\mathrm{it}}=\frac{{\mathrm{ROA}}_{\mathrm{it}}+{\mathrm{EQA}}_{\mathrm{it}}}{\mathrm{sd}{\left(\mathrm{ROA}\right)}_{\mathrm{it}}}$$

Where: **ROA** represents the return on assets, **EQA** denotes the ratio between bank’s total equity and total assets and **Sd** stands with standard deviation.

**3.2.2 Independent variables**

Measuring FI is essential to support evidence-based policy decisions ( Sarma, 2016; Nguyen, 2021). Accurate measurement reveals gaps and opportunities for expanding FI ( Demirgüç-Kunt et al., 2020). However, relying on a single factor can be misleading ( Sarma, 2008; Nguyen, 2021). Composite indices offer a more comprehensive view by integrating multiple dimensions, enabling better comparisons across time and regions ( OECD, 2008). These indices capture the multifaceted nature of FI (Mishra, 2007). This study constructs a multidimensional FI index using two-stage PCA, combining10 convensional indicators and 5 digital indicators (see Table 1).

**Table 1.** Overview of indicators and data sources used in the study.

Variables	Notation	Definition	Sources	Studies
***Bank stability (Dependent variable)***				
Bank Z-Score	ZS	Computes the buffer of a state’s banking system with the volatility of those returns.	NBE	Damrah et al., 2023; Koudalo & Toure, 2023; Barik & Pradhan, 2021; Sethy & Goyari, 2022; Vo et al., 2020; Ahamed & Mallick, 2019
***Financial inclusion indicators (Independent variable**)*				
Convensional Availability	BPC	No of Branches (per 100,000 adults)	NBE; WB	Damrah et al., 2023; Hua et al., 2023; Gharbi & Kammoun, 2023; Jima & Makoni, 2023; Jungo et al., 2022; Barik & Pradhan, 2021; Ismael & Ali, 2021; Kebede et al., 2021; Nguyen, 2021; Sha’ban et al., 2020; Vo et al., 2020; Le et al., 2019; Ahamed & Mallick, 2019; Camara & Tuesta, 2017
Convensional Availability	BAC	No of Branches (per 1000 km ^2^)	NBE; WB	
Convensional Availability	APC	No of ATMs (per 100,000 adults)	NBE; WB	
Convensional Availability	AAC	No of ATMs (per 1000 km ^2^)	NBE; WB	
Convensional Availability	PPC	No ofPoSs (per 100,000 adults)	NBE; WB	
Convensional Availability	PAC	No ofPoSs (per 1000 km ^2^)	NBE; WB	
Digital Availability	APD	No of agents (per 1,000 adults)	NBE; WB	
Digital Availability	AAD	No of agents (per 1,000 km ^2^)	NBE; WB	
Digital Accessibility	MPD	No of mobile active users (per 1,000 adults)	NBE; WB	
Digital Accessibility	IPD	No of internet active users (per 1,000 adults)	NBE; WB	
Digital Accessibility	WPD	No of mobile money (wallet) users (per 1,000 adults)	NBE; WB	
Convensional Usage	DPC	Depositors with banks (per 1,000 adults)	CBE; WB	
Convensional Usage	APC	No of debit cards per 1,000 adults.	NBE; WB	
Convensional Usage	LGDPC	Outstanding loans and advances (% of GDP)	NBE	
Convensional Usage	DGDPC	Outstanding deposits (% of GDP)	NBE	
***Control variables***				
Loan to Deposit ratio	LDR	Indicates how much of the bank's deposit base is being used for lending.	NBE	Muhammed et al., 2024; Kulu & Bondzie, 2024; Bod’a & Zimková, 2021; Hakim, 2017
Provision to Loan	PL	The loan loss provision ratio represents the funds set aside to cover expected credit losses.	NBE	Ha & Nguyen, 2023; Hua et al., 2023; Koudalo & Toure, 2023; Barik & Pradhan, 2021; Ahamed & Mallick, 2019; Chen et al., 2019
Natural logarithm of Total Asset	lnTA	Provides a comprehensive measure of the bank's financial strength and capacity, reflecting the aggregate value of its economic resources available for generating revenue and supporting operations.	NBE	Damrah et al., 2023; Jungo et al., 2022; Ramzan et al., 2021; Ahamed & Mallick, 2019; Vo et al., 2020; Ali & Puah, 2018; Beck et al., 2013a; Gupta & Kashiramka, 2020
Capital adequacy ratio	CAR	Assesses a bank's ability to absorb potential losses and sustain operations during financial stress, ensuring it maintains sufficient capital to cover its risks and support continued stability	NBE	Hua et al., 2023; Jungo et al., 2022; Kebede et al., 2021; Anarfo et al., 2020; Vo et al., 2020
Income Diversification	IND	The non-interest income to total income ratio insight into the diversification of a bank's income streams beyond traditional interest-based revenue.	NBE	Ahamed & Mallick, 2019; Sanya & Wolfe, 2011; Elsas et al., 2010
Operational efficiency management	EF	It refers to the practices and strategies employed to optimize a bank's operational processes, reduce costs, and enhance productivity.	NBE	Damrah et al., 2023; Ullah et al., 2023; Jungo et al., 2022; Ahamed & Mallick, 2019; Beck et al., 2013
Real lending interest rate	RLIR	Inflation adjusted lending interest rate, i.e., lending interest rate minus inflation rate	NBE	Koudalo & Toure, 2023; Anthony-Orji et al., 2022; Atellu & Muriu, 2022; Siddik & Kabiraj, 2018
GDP Growth Rate	GDP	Annual percentage change in gross domestic product	NBE	Damrah et al., 2023; Ha & Nguyen, 2023; Koudalo & Toure, 2023; Oanh et al., 2023; Sethy & Goyari, 2022; Vo et al., 2020; Beck et al., 2013

Source: Prepared by authors.

**3.2.3 Control variables**

As outlined in Table 1, the study includes several bank-specific and macroeconomic control variables, including an index for operational efficiency. Non-parametric index is used to drive efficiency using an output-oriented Constant Returns to Scale (CRS) through Data Envelopment Analysis (DEA). The choice of CRS is justified by the structure of the Ethiopian banking industry. Ayalew & Xianzhi (2017) highlight that Ethiopian banks operate within a homogeneous regulatory framework with limited competition. Most banks in Ethiopia are subject to similar capital requirements, operational constraints, and government policies, which result in a uniform relationship between inputs and outputs across banks. Despite Rao & Lakew (2012) suggesting that managers in Ethiopian banks have more control over inputs than outputs, most banks prioritize maximizing profits through efficient use of inputs. Thus, assuming out-put oriented CRS provides a realistic framework for comparing efficiency, as banks are expected to scale their operations proportionally regardless of size, making it suitable for long-term efficiency assessments.

In this study, salary and benefits, provisions, general expenses, branches, and deposits are treated as inputs, while net interest income and non-interest income serve as outputs. Using the model by Charnes et al. (1978) under CRS, the efficiency score θ is calculated as the ratio of weighted sum of outputs to inputs.

$$\text{Efficiency}\;\left(\theta \right)=\frac{\sum_{r=1}^{s}u_{r}Y_{r_{j}}}{\sum_{i=1}^{m}v_{i}X_{i_{j}}}\leq 1,j=1,2,\ldots ,n,$$

Where (θ) = efficiency score of bank j , $Y_{r_{j}}$ = amount of output r produced by bank j , $X_{i_{j}}$ = amount of input i used by bank j , $u_{r}$ = weight assigned to output r , $v_{i}$ = weight assigned to input i , s = number of outputs, m = number of inputs, n = number of banks (decision-making units) being evaluated.

Additionally, the analysis includes control variables such as income diversification, capital adequacy ratio, provisions to non-performing loans, loan-to-deposit ratio, and total assets. Macroeconomic variables include the real lending interest rate and GDP growth rate.

### 3.3 Financial inclusion composite index development strategy

PCA is employed to construct an index that effectively reduces data dimensionality while preserving much information ( Tram et al., 2023; Nguyen, 2021; Hair et al., 2019; Sha’ban et al., 2020; Camara & Tuesta, 2017). Despite non-parametric approaches like Sarma (2008) model, which often rely assiging weightson researcher’s experience ( Tram et al., 2023; Camara & Tuesta, 2017), PCA adapts to changes in data structure ( Decancq & Lugo, 2013), avoiding the pre-assignment of weights before data collection ( Chen et al., 2019). Additionally, compared to parametric methods like Confirmatory Factor Analysis (CFA), PCA enhances objectivity by not requiring subjective decisions on factor structure ( Tram et al., 2023; Camara & Tuesta, 2017; Ismael & Ali, 2021; Jolliffe & Cadima, 2016; Steiger, 1979), which may differ across time and space.

Normalization is essential for comparing indicators with varying units and ranges. Common methods include ranking, z-score standardization, min-max rescaling, and logarithmic transformation ( Carrino, 2015; Freudenberg, 2003; OECD, 2008). In this study, the min-max ( MMY ) approach is used to normalize indicators to a scale of 0 to 1, where 0 represents exclusion and 1 represents inclusion.

$$\mathrm{MMY}=\frac{Y_{i}-Y_{\min}}{Y_{\max}-Y_{\min}},$$

Where $Y_{\min}$ , minimum value, $Y_{\max}$ , maximum value

Traditional single-level multivariate analysis often fails to address nested data structures due to its assumption of independent and identically distributed (i.i.d) observations, potentially missing within groupinformation ( Yang et al., 2022). To mitigate this, a multi-level framework is employed, which addresses the hierarchical nature of the data, enhancing analytical precision ( Yang et al., 2022; Hox, 2013). Additionally, single-stage PCA may disproportionately weight indicators with unequal variances. By generating sub-indices within each dimension separately before combining them, the multilevel approach provides a more balanced and accurate composite index ( Ismael & Ali, 2021; Camara & Tuesta, 2017; Nagar & Basu, 2002).

**3.3.1 First-stage analysis**

Table 2 shows the results of the first-stage PCA. To ensure the suitability of PCA, the Kaiser–Meyer–Olkin (KMO) measure and Bartlett’s test of sphericity are performed. These tests confirm that the sample size is adequate and the indicators are sufficiently intercorrelated ( Ismael & Ali, 2021; Taherdoost et al., 2014). A KMO value of 0.5 or higher and a statistically significant chi-square in Bartlett’s test (1950) indicate that factorization is appropriate ( Hair et al., 2019). As reported in Table 2, all components meet these criteria (KMO ≥ 0.5 and p-value < 0.05), supporting the use of PCA for FI index development. Additionally, internal consistency was measured using a reliability test. According to Kline (2015), a Cronbach’s alpha value above 0.6 is acceptable for developing scales. The obtained Cronbach’s alpha of 0.9721, as shown in Table 2, exceeds this threshold, indicating excellent internal consistency and enhancing the reliability of the data.

**Table 2.** First stage PCA result.

Estimation of PC and eigenvalue of sub-indices of TFI and DFI			
Component	Eigenvalue	Difference	Explained variance
Availability TFI			
Comp1	5.4682	5.1268	0.9114
Comp2	0.3415	0.1566	0.0569
Comp3	0.1849	0.1801	0.0308
Comp4	0.0048	0.0043	0.0008
Comp5	0.0004	0.0002	0.0001
Comp6	0.0003		0
Availability DFI			
Comp1	1.9997	1.9994	0.9999
Comp2	0.0003		0.0001
Accessibility DFI			
Comp1	2.0686	1.2765	0.6895
Comp2	0.7921	0.6528	0.264
Comp3	0.1373		0.0464
Usage DFI			
Comp1	3.4725	2.992	0.8681
Comp2	0.4804	0.443	0.1201
Comp3	0.0374	0.0278	0.0094
Comp4	0.0096		0.0024

Scoring coefficients for orthogonal varimax rotation (weights) and test results							
Dimensions	Indicators	Comp1	Unexplained variance	Normalized weights	KMO	Sphericity test	Cronbach alpha
Convensional availability	BP	0.4146	0.06	0.16927	0.6974	*χ* ^2^(15) = 3347.798 (P = 0.000)	0.9721
	BA	0.4105	0.0786	0.16759	0.6875		
	AP	0.4112	0.0753	0.1679	0.6929		
	AA	0.4078	0.0908	0.16649	0.6851		
	PP	0.3983	0.1324	0.16263	0.6931		
	PA	0.4069	0.0948	0.16612	0.7104		
Digital availability	AP	0.7071	0.0001	0.5	0.5	*χ* ^2^(1) = 1,119.314 (P=0.000)	
	AA	0.7071	0.0001	0.5	0.5		
Digital Accessibility	MP	0.6382	0.1574	0.375736	0.537	*χ* ^2^(3) = 221.805 (P = 0.000)	
	IP	0.4067	0.6578	0.239446	0.7997		
	WP	0.6536	0.1162	0.384818	0.5332		
Convensional usage	DP	0.4691	0.236	0.234706	0.5593	*χ* ^2^(6) = 1,111.108 (P = 0.000)	
	AP	0.5195	0.0627	0.259963	0.6725		
	LGDP	0.4978	0.1396	0.249065	0.5795		
	DGDP	0.5121	0.0892	0.256266	0.654		

Source: Computed based on Stata 14 result.

Hair et al. (2019) deem an explained variance (EV) of 60% or higher as acceptable. Table 2 shows that PC1 captures a significant portion of the variance in the availability and usage dimensions (91.1% and 86.8%, respectively), confirming their substantial role in FI index development. For digital inclusion, PC1 explains nearly 99.9% of the variance in availability, indicating its dominance. As well, PC1 explains 69% of the variance in accessibility, highlighting that while both dimensions are important, they contribute differently to the FI index. Based on Table 2 first-stage PCA result, the following result is built for each indicators, The dimensions of “availability”, and “usage” of the Convensional sub-index is;

$${\text{CAvailability}}_{\mathrm{it}}=0.1693{\mathrm{BPC}}_{\mathrm{it}}+0.1676{\mathrm{BAC}}_{\mathrm{it}}+0.1679{\mathrm{APC}}_{\mathrm{it}}+0.1665{\mathrm{AAC}}_{\mathrm{it}}+0.1626{\mathrm{PPC}}_{\mathrm{it}}+0.1661{\mathrm{PAC}}_{\mathrm{it}}+\upsilon_{\mathrm{it}}$$

$${\text{CUsageC}}_{\mathrm{it}}=0.2347{\text{DCPC}}_{\mathrm{it}}+0.26{\mathrm{APC}}_{\mathrm{it}}+0.2491{\text{DGDPC}}_{\mathrm{it}}+0.2563{\text{LGDPC}}_{\mathrm{it}}+\varepsilon_{\mathrm{it}}$$

The analysis reveals that branch availability has the highest relative weight compared to ATMs and PoS machines in both demographic and geographic contexts, emphasizing the crucial role of physical branches in Ethiopia. This finding supports Kebede et al. (2021) and highlights the continued importance of physical branches due to factors such as lower financial literacy, which necessitates in-person interactions. Within the usage dimension, deposit accounts (0.26) hold the highest weight, followed by the deposit to GDP ratio (0.2563), loan to GDP ratio (0.2491), and debit card penetration (0.2347). This underscores the current focus on deposit mobilization in Ethiopia, which collectively emphasize the sector's drive to encourage savings. The dimensions of “Availability”, and “Accessibility” of the digital sub-index is given as follows:

$$D{\text{AvailabilityD}}_{\mathrm{it}}=0.5{\mathrm{APD}}_{\mathrm{it}}+0.5{\mathrm{AAD}}_{\mathrm{it}}+{\text{€}}_{\mathrm{it}}$$

$${\text{DAccessibilityD}}_{\mathrm{it}}=0.3757{\text{MBPD}}_{\mathrm{it}}+0.2395{\text{IBPD}}_{\mathrm{it}}+0.3848{\mathrm{WPD}}_{\mathrm{it}}+{\text{€}}_{\mathrm{it}}$$

In digital availability, agency banking indicators (APD and AAD) have equal weights of 0.5, reflecting their equal importance. For digital accessibility, mobile money wallets have the highest weight (0.3848), followed by mobile banking penetration at 0.3757 and internet banking penetration at 0.2394. This indicates that mobile money is the preferred digital financial access method for the general population, contrasting with internet banking, which is primarily used by elitecustomers. This finding aligns with the observation that Ethiopian banks focus internet banking services on premium clients.

**3.3.2 Second-stage analysis**

Table 3 confirms that the KMO measure exceeds the recommended threshold of 0.5 ( Hair et al., 2019), and Sphericity test is highly significant (p-value < 0.0001). The first principal component accounts for 80.07% of the total variance, indicating that only 19.97% of the variance remains unexplained. This result confirms the adequacy of the PCA extraction, as supported by Hair et al. (2019). Among four components, only one has an eigenvalue greater than 1, which is retained for constructing the FII. The derived FII is expressed as follows:

$$\mathrm{IFI}=0.2625Y_{i}^{a_{t}}+0.2616Y_{i}^{u_{t}}+0.2254Y_{i}^{a_{d}}+0.2505Y_{i}^{p_{d}}+{℮}_{i}$$

**Table 3.** Second-stage PCA result.

Estimation of PC and eigenvalue of sub-indices of TFI and DFI				
Component	Eigenvalue	Difference	Proportion	Cumulative
Comp1	3.20293	2.67426	0.8007	0.8007
Comp2	0.528673	0.281266	0.1322	0.9329
Comp3	0.247407	0.226421	0.0619	0.9948
Comp4	0.0209866		0.0052	1

Scoring coefficients for orthogonal varimax rotation (weights) and test results					
Indicators	Comp1	Unexplained	Normalized weights	KMO	Sphericity test
Availability	0.524	0.1205	0.2625	0.5948	*χ* ^2^(6) = 709.297 (p = 0.000)
Usage	0.5223	0.1263	0.2616	0.6121	
Availability	0.45	0.3515	0.2254	0.6663	
Accessibility	0.5002	0.1987	0.2505	0.6991	

Source: Computed based on Stata 14 result.

This equation indicates that Convensional availability (0.2625) has the highest weight, underscoring the critical role of infrastructure development in enhancing FI. This aligns with findings from Nguyen (2021) and Gharbi and Kammoun (2023), emphasizing the need for robust financial infrastructure alongside efforts in financial literacy and user adoption. Usage (0.2616) is the second most significant dimension, reflecting the importance of account usage in FI. Digital accessibility (0.2505) ranks third, highlighting the increasing impact of digital tools. Agency banking, with the lowest weight (0.2254), suggests that while digital solutions are cost-effective, more targeted interventions are required to address the needs of vulnerable populations, including women, rural residents, and those with lower literacy levels.

To verify the strength of the researchers used the overall average of FI of the country and measured the correlation between the saving and real lending interest rate and newly developed FI index. The Pearson correlation results presented in Table 4 denotes p-values of 0.0000 and 0.0002, respectively, represents that the findings are significant at 1% level (0.9617 for saving) and (-0.9391 for real lending interest rate). The direction of the correlations aligns with theoretical expectations. Real lending interest rates are negatively correlated with FI, as high rates make loans more expensive and discourage access, particularly for low-income populations ( Gharbi & Kammoun, 2023; Uddin et al., 2017). Conversely, in relation to deposits, a positive higher FI scores are associated with in-creased savings, likely due to a greater number of households having access to financial institutions and associated saving products ( Gharbi & Kammoun, 2023). These robust correlations provide strong evidence for the construct validity of the newly developed FI index.

**Table 4.** Pearson correlation between FI index, saving and real deposit interest rate.

		FII
FII	Pearson Correlation	1
Saving	Pearson Correlation	0.9617***
	Sig. (bilateral)	0.0000
RLIR	Pearson Correlation	-0.9391***
	Sig. (bilateral)	0.0002

Source: Computed based on Stata 14 result.

### 3.4 Model specification

This study adopts an empirical exploration, employing a panel methodology, to delve into the crossectional and longitudinal dynamics of evaluating the impact of FI on bank stability —within the period 2015-2023. The empirical model for examining the impact of FI on bank stability, the researchers estimated this dynamic panel model:

$${\mathrm{ZS}}_{\mathrm{it}}=\beta_{0}+\beta_{1}{\mathrm{IFI}}_{\mathrm{it}}+\beta_{2}{\mathrm{LDR}}_{\mathrm{it}}+\beta_{3}{\mathrm{PL}}_{\mathrm{it}}+\beta_{4}{\text{lnTA}}_{\mathrm{it}}+\beta_{5}{\mathrm{CAR}}_{\mathrm{it}}+\beta_{6}{\mathrm{EF}}_{\mathrm{it}}+\beta_{7}{\mathrm{IND}}_{\mathrm{it}}+\beta_{8}{\text{RLIR}}_{\mathrm{it}}+\beta_{9}{\mathrm{GDP}}_{\mathrm{it}}+\varepsilon_{\mathrm{it}}$$

Where, ${\mathrm{ZS}}_{\mathrm{it}}$ is the measure of bank stability, represented by Z-score, ${\mathrm{IFI}}_{\mathrm{it}}$ , the index of FI extracted from two staged PCA, ${\mathrm{LDR}}_{\mathrm{it}}$ , represents the loan to deposit ratio, ${\mathrm{PL}}_{\mathrm{it}}$ , refers to provision to loan ratio, ${\text{lnTA}}_{\mathrm{it}}$ , indicates the natural logarithm of total asset, ${\mathrm{CAR}}_{\mathrm{it}}$ , denotes risk weighted capital adequacy ratio, ${\mathrm{EF}}_{\mathrm{it}}$ , refers to the efficiency ratio extracted from DEA approach, ${\mathrm{IND}}_{\mathrm{it}}$ , refers to non interest income to total income ratio, ${\text{RLIR}}_{\mathrm{it}}$ , represents inflation adjusted lending interest rate, ${\mathrm{GDP}}_{\mathrm{it}}$ , refers to the gross domestic product growth rate, $\varepsilon_{\mathrm{it}}$ , refers error term, $\beta_{0}$ , refers to the constant term,and $\beta_{1\;\text{to}\;9}$ , represents the slope of the independent variables. *i* = 1.. N & t = 1.. T, denotes to cross-section & time, respectivelly.

## 4. Results and Discusion

### 4.1 Results

**4.1.1 Descptive analysis**

Table 5 presents the descriptive summary for the variables used to examine the impact of financial FI on bank stability. The analysis is based on a sample of 153 observations, covering 8 years (2015-2023) across 17 commercial banks (all (17) commercial banks operationalized since 2015 are taken for further analysis). The table provides key details, including the mean, standard deviation, and the minimum and maximum values for each variable.

**Table 5.** Summary analysis.

Variable	Obs	Mean	Std. Dev.	Min	Max
ZS	153	32.403	15.712	8.972	80.117
IFI	153	0.059	0.112	0.002	0.584
LDR	153	0.963	0.089	0.533	1.159
PL	153	0.007	0.010	-0.001	0.087
lnTA	153	10.155	1.320	7.042	14.082
CAR	153	0.188	0.089	0.084	0.958
EF	153	0.893	0.098	0.624	1.000
IND	153	0.284	0.116	0.006	0.570
RLIR	153	-0.036	0.083	-0.196	0.056
GDP	153	0.079	0.015	0.061	0.104

Source: Computed based Stata 14 result.

**4.1.2 Empirical analysis technique**

**4.1.2.1 *Analysis of ordinary least-square regression (OLS)***

Using fixed or random effects models in OLS regression is common for addressing unobserved heterogeneity and simultaneous causality in panel data ( Kumar et al., 2022). Fixed effects handle time-invariant characteristics within firms ( Schultz et al., 2010), while random effects reduce variability by pooling data and accounting for differences across firms and time ( Kumar et al., 2022). The Hausman test, which checks for the correlation between unique errors and regressors, guides the choice between these models ( Kumar et al., 2022; Greene, 2003). Our results indicate that the fixed-effects model is the correct choice, as shown in Table 9.

To test for autocorrelation and heteroskedasticity, the Wooldridge, modified Wald, and Breusch-Pagan (BP) tests were employed. Results in Table 6 reveals, both problems were detected. However, the correlation matrix and Variance Inflation Factor (VIF) results in Table 7, with all VIF values lessthan 10, suggest that multicollinearity is not a concern. Following Vo et al. (2020) and Ahamed and Mallick (2019), robust clustered standard errors were applied to address these issues, as outlined in Table 9.

**Table 6.** Heteroscedasticity and autocorrelation test.

Tests	Wooldridge test	Modified Wald test	Breusch-Pagan (BP) test
Type	Serial correlation	Heteroscedasticity	Heteroscedasticity
	F-test	Chi ^2^ test	Chi ^2^ test
Sig level	*F(1,16)=30.970 (p=0.0000)*	*χ ^2^(17)=112.98 (p=0.0000)*	*χ ^2^(1)=7.54 (p=0.0060)*

Source: Computed based on Stata 14 result.

**Table 7.** Multicollinearity test.

Variables	VIF	1/VIF	ZS	IFI	LDR	PL	TA	CAR	EF	IND	RLIR
IFI	3.94	0.254	-0.29								
LDR	1.43	0.701	-0.02	0.423							
PL	1.12	0.894	-0.35	0.262	0.215						
lnTA	5.69	0.176	-0.16	0.718	0.364	0.220					
CAR	1.26	0.791	-0.1	0.045	-0.125	-0.007	-0.209				
EF	1.36	1.3	0.771	0.203	0.323	0.082	0.202	0.012			
IND	2.41	0.414	-0.07	-0.253	-0.282	-0.076	-0.647	0.417	-0.039		
RLIR	3.03	0.33	0.071	0.000	-0.160	-0.138	-0.462	0.255	-0.308	0.446	
GDP	2.32	0.431	0.071	0.000	-0.170	-0.112	-0.395	0.230	-0.195	0.460	0.736

Source: Computed based on Stata 14 result.

**4.1.3 GMM analysis**

**4.1.3.1 Stationary test**

Conducting a unit root test is crucial to confirm stationarity of variables, thereby avoiding the risk of spurious findings ( Breitung & Pesaran, 2005). Although a dynamic panel approach is typically suited for variables integrated at level or first difference, it is crucial to ensure none of the variables is integrated at a higher order, such as I(2) ( Pesaran & Smith, 1995). To verify stationarity, we employed the Levin-Lin-Chu (LLC) and Harris-Tzavalis (HT) unit root tests across both I(0) and I(1) levels, as shown in Table 8. The LLC test confirms that all variables, are stationary at level. Similarly, the HT test shows that only total assets achieve stationarity at first difference. Since all variables are either stationary at level or first difference, applying the GMM method is appropriately validated.

**Table 8.** Stationary test result: LLC and HT.

	LLC		HT	
Variables	T-statistics	Order	T-statistics	Order
ZS	(7.1564) ***	I(0)	0.5428 *	I(0)
IFI	(6.7882) ***	I(0)	(0.0528) ***	I(0)
LDR	(3.7723) ***	I(0)	0.1663 ***	I(0)
PL	(17.1597) ***	I(0)	0.1221 ***	I(0)
lnTA	(6.7186) ***	I(0)	0.0677 ***	I(1)
CAR	(8.2427) ***	I(0)	(0.0436) ***	I(0)
EF	(2.9716) **	I(0)	0.425 ***	I(0)
IND	(3.8704) ***	I(0)	(0.2079) ***	I(1)
RLIR	(8.5431) ***	I(0)	(0.0852) ***	I(0)
GDP	(5.8930) ***	I(0)	0.2165 ***	I(0)

Source: Computed based on Stata 14 result.

* p < 0.05.

** p < 0.01.

*** p < 0.001.

Traditional FE and RE models, while valuable, often struggle to address endogeneity concerns stemming from omitted variables, measurement errors, reverse causality, and time-invariant unobserved heterogeneity in panel data ( Hill et al., 2020; Leszczensky & Wolbring, 2022). These issues are exacerbated by the presence of lagged dependent variables and endogenous regressors, leading to biased and inconsistent estimates ( Leszczensky & Wolbring, 2022; Bellemare et al., 2017). In the context of dynamic panel data, particularly “small T, large N” panels, selecting an appropriate estimation technique is critical for obtaining reliable and efficient results ( Roodman, 2009)

The Arellano and Bond (1991) estimator is particularly effective in addressing dynamic dependent variables and non-strictly exogenous regressors. By differencing the regressors to eliminate fixed effects and applying GMM, it mitigates potential biases ( Roodman, 2009). The system GMM estimator, introduced by Arellano & Bover (1995) and Blundell & Bond (1998), extends this by assuming the first differences of the instruments are uncorrelated with fixed effects, thus incorporating additional instruments and improving estimation efficiency ( Jima & Makoni, 2023; Bun & Windmeijer, 2010). System GMM constructs a two-equation system, original levels and transformed differences, leveraging both levels and differences of the instruments ( Roodman, 2009). This approach effectively addresses dynamic panel bias, which often distorts small-sample properties and yields unstable estimates when varied instrument sets are employed ( Blundell & Bond, 2023; Windmeijer, 2005).

As noted by Morgan and Pontines (2017), system GMM not only addresses endogeneity bias but also remains consistent and efficient in the presence of heteroskedasticity and autocorrelation within individuals. Our results, as presented in Table 6, confirm the existence of heteroskedasticity and autocorrelation within the model. The system GMM approach is particularly effective in addressing serial correlation and unobserved heterogeneity ( Vo et al., 2020; Roodman, 2009). Therefore, employing GMM to overcome these issues aligns with previous empirical studies ( Jima & Makoni, 2023; Vo et al., 2020; Ahamed & Mallick, 2019; Morgan & Pontines, 2017).

Blundell et al. (2001) and Bond et al. (2001) highlight significant improvements when dealing with models that include a lagged dependent variable along with other explanatory variables. To tha end, this study refines the previous model into a dynamic form, ensuring more robust and consistent estimations. The specified System-GMM model is as follows:

$${\mathrm{ZS}}_{\mathrm{it}}=\alpha_{i}+\mathrm{\varphi i}.{\mathrm{ZS}}_{i,t-1}+\sum_{1}^{n}\beta_{\mathrm{it}}X_{\mathrm{it}}+\in_{\mathrm{it}}$$

In this equation, ${\mathrm{ZS}}_{\mathrm{it}}$ represents the dependent variable, $\alpha_{i}$ is the individual-specific effect, φ is the coefficient for the lagged dependent variable, $\beta_{it}$ are the coefficients for the explanatory variables $X_{it}$ , and $\in_{it}$ is the error term.

As per the works established by Bond et al. (2001), Bond (2002), and Presbitero (2006, 2008), the System GMM estimator was selected based on a comparative analysis of autoregressive( φ ) coefficients derived from Pooled OLS, Fixed Effects, and Difference GMM estimations. Following Bond et al.'s (2001) recommendation, the Pooled OLS coefficient served as an upper bound, while the Fixed Effects coefficient acted as a lower bound. Given that the Difference GMM coefficients (0.347 and 0.328) were significantly lower than the Fixed Effects coefficient (0.412) and well below the Pooled OLS coefficient (0.947), as presented in Table 9, the System GMM approach was deemed appropriate.

**Table 9.** Empirical result of the study.

Variables	Robust Fixed Effect	Robust Pooled OLS	Robust One-step Difference GMM	Robust Two-step Difference GMM	Robust One-step System GMM	Robust Two-step System GMM
l.ZS	0.412 ^***^	0.947 ^***^	0.347 ^**^	0.328	0.798 ^*^	1.155 ^***^
	(5.62)	(55.49)	(2.15)	(1.15)	(2.08)	(3.07)
IFI	-8.811	-2.890	-10.41	-9.382	49.93 ^**^	84.07 ^**^
	(-0.99)	(-0.52)	(-1.00)	(-0.65)	(2.85)	(2.60)
LDR	10.82 ^***^	1.010	11.51 ^***^	9.283	-19.10	-55.46
	(3.43)	(0.26)	(2.95)	(1.74)	(-1.07)	(-1.73)
PL	-69.73 ^**^	-63.76 ^**^	-70.40 ^**^	-63.67 ^*^	-125.7	17.66
	(-2.71)	(-2.27)	(-2.77)	(-1.96)	(-0.42)	(0.07)
lnTA	-2.249 ^**^	0.321	-2.342 ^**^	-1.937 ^*^	-8.085 ^***^	-10.03 ^**^
	(-2.30)	(0.65)	(-2.39)	(-1.83)	(-3.04)	(-2.66)
CAR	3.488	0.747	3.566	1.414	10.68	-37.80
	(0.71)	(0.45)	(0.69)	(0.18)	(0.41)	(-0.99)
EF	13.61 ^***^	7.436 ^**^	14.04 ^***^	13.21 ^*^	15.35	26.24 ^*^
	(3.56)	(2.60)	(3.65)	(2.00)	(1.46)	(2.00)
IND	6.825 ^*^	1.230	7.208 ^*^	5.309	-71.81	-101.7 ^*^
	(1.80)	(0.41)	(1.80)	(1.09)	(-1.39)	(-1.94)
RLIR	-11.38	-6.605	-10.55	-6.237	-32.19 ^*^	-33.46
	(-1.54)	(-1.26)	(-1.41)	(-0.64)	(-1.99)	(-1.42)
GDP	9.593	29.75	7.668	4.417	105.3	229.5 ^*^
	(0.42)	(1.05)	(0.35)	(0.19)	(1.38)	(2.12)
_cons	16.42 ^*^	-12.12			99.98 ^**^	138.4 ^**^
	(1.86)	(-1.58)			(2.92)	(2.44)
*N*	136	136	119	119	136	136
*Groups*	17		17	17	17	17
*Instruments*	-	-	16	16	16	16
*AR(2) test*	-	-	-1.46 (0.144)	-1.38 (0.167)	-0.12 (0.908)	-0.71 (0.479)
*Hansen test*	-	-	10.55 (0.103)	10.55 (0.103)	4.12 (0.532)	1.13 (0.951)
*R-Squared *	0.7632	0.9665				
*P-Value *	F(10,16) = 68.14 (p=0.000)	F(10,125) = 413.07 (p=0.000)	F(10,17) = 13.59 (p=0.000)	F(9,143) = 8.49 (p=0.000)	F(10,16) = 2104.9 (p=0.000)	F(10,16) = 592.07 (p=0.000)

Source: Computed based on stata 14 result.

^*^ *p*< 0.1.

^**^ *p*< 0.05.

^***^ *p*< 0.01.

The System GMM estimator enhances robustness and efficiency by using both difference and level moment conditions to address endogeneity and ensure reliable inference ( Sebki, 2021; Soto, 2009; Presbitero, 2008). To handle heteroscedasticity and serial correlation, a two-step System GMM procedure with a consistent weighting matrix from one-step residuals was used ( Davidson & MacKinnon, 2004). Although two-step GMM estimates can have downward-biased standard errors ( Presbitero, 2008), the small-sample correction by Windmeijer (2005) was applied to improve efficiency, making it preferable over one-step robust GMM ( Roodman, 2006). Consequently, the two-step robust System GMM estimator was thus selected for further analysis. To prevent overfitting, the maximum number of instrument lags was constrained. The AR(2) and Hansen tests were conducted to validate the absence of misspecification and the appropriateness of the instrumental variables in the GMM estimation.

**4.1.4 Causality test**

This research examines the dynamic causal relationship between FI and bank stability within the Ethiopian banking sector. Both the Dumitrescu and Hurlin (2012) W-statistic test and the Juodis et al. (2021) Z-bar test are used to ascertain the direction of causality between FI and bank stability. Rejection of the null hypothesis indicates a causal relationship, whereas its acceptance suggests none ( Antwi et al., 2024). The analysis employs a first lag, with lag selection based on the Modified Bayesian Information Criterion (MBIC) to ensure result robustness, in line with Hussain et al. (2024) and Žiković et al. (2020) recommendations for shorter time periods.

### 4.2 Discusion

The findings reveal that the lagged value of bank stability positively effect current stability. Specifically, past levels of bank stability significantly impact present stability, as corroborated by the study of Yitayaw et al. (2023) within the Ethiopian context. The positive effect of lagged stability on current conditions underscores its validity as a reliable instrument for stability analysis, given that banks generally exhibit continuity in their stability across different periods, reflecting a trend of persistence over time.

Our findings confirm a significant positive relationship between FI and bank stability, supported by existing research ( Koudalo & Toure, 2023; Nguyen & Du, 2022; Vo et al., 2020; Le et al., 2019; Neaime & Gaysset, 2018; Morgan & Pontines, 2014). Specifically, a 1% increase in FI is associated with an 84.07 unit increase in bank stability in the short run, ceteris paribus. Enhanced FI strengthens bank stability by diversifying bank assets, reducing systemic and liquidity risks, and improving monetary policy transmission ( Ahamed & Mallick, 2019; Morgan & Pontines, 2014; Khan, 2011; Hannig & Jansen, 2010). It also addresses information asymmetry by allowing lenders to better assess borrowers and by providing banks with critical proprietary information ( Nguyen & Du, 2022), thus fostering a more resilient banking sector.

Our findings indicate that control variables such as lnTA, EF, IND, and GDP growth rate significantly affect bank stability. Larger banks exhibit decreased stability, with a 10.03 unit decline per percentage increase in total assets in the short run, ceteris paribus. This may be due to increased operational and systemic risks associated with larger institutions, aligning with Vo et al. (2020) and Mu and Lin (2016), but differing from Nguyen and Du (2022) and Ahamed and Mallick (2019). Efficiency positively effect stability, with a 1% increase in efficiency leading to a 26.24 unit rise in stability in the short run, ceteris paribus. This suggests that higher efficiency improves profitability and stability, consistent with Nguyen and Du (2022), and Ahamed and Mallick (2019). Income diversification negatively impacts stability, with a 1% increase resulting in a 101.7 unit decrease in stability in the short run, ceteris paribus. This might reflect the risks introduced by diversification, aligning with Nguyen and Du (2022) but differing from Ahamed and Mallick (2019). In the Ethiopian context, where banks are predominantly dependent on deposit revenue, an abrupt or poorly managed shift towards greater non-interest income might contribute to instability. GDP growth rate shows a positive effect on stability, with a 1% increase leading to a 229.5 unit rise in stability in the short run, ceteris paribus. This suggests that economic growth enhances bank stability, consistent with Nguyen and Du (2022), Vo et al. (2020), and Ahamed and Mallick (2019).

The two-step System GMM analysis, as detailed in Table 9, shows that the standard diagnostic tests are supportive of the model’s validity. The AR(2) test (Prob > χ ^2^ = 0.479) does not reject the null hypothesis, indicating no evidence of second-order residual autocorrelation. Similarly, the Hansen test (Prob > χ ^2^ = 0.951) confirms the validity of the instruments, verifying their appropriateness. The F-test (Prob > F = 0.000) demonstrates a strong goodness of fit for the model. In addition to the two-step robust System GMM results, findings from robust FE, robust Pooled OLS, robust one-step Difference GMM, robust two-step Difference GMM, and robust one-step System GMM are largely consistent. The main variable, FI, is significant in both one-step and two-step System GMM models, while the GDP growth rate is significant in the two-step robust System GMM. Overall, these results underscore the robustness of the employed models.

In addition, based on the result revealed in Table 10, the Juodis et al. (2021) test confirms that FI significantly Granger-causes bank stability at the 5% level, a result supported by the Dumitrescu and Hurlin (2012) test, which also rejects the null hypothesis. This underscores the substantial role of FI in bolstering the resilience and stability of Ethiopia's banking sector. Additionally, reverse causality from bank stability to FI is significant at the 5% level, indicating that bank stability can predict enhancements in FI. These findings suggest that enhancing FI can improve bank stability, while a stable financial environment fosters greater FI, aligning with previous studies ( Jima & Makoni, 2023; Antwi et al., 2024).

**Table 10.** Causality test result.

Test	Z-bar	P-Value	Direction	Conclusion
ZS≠IFI	7.3252 ***	95.3201 ***	ZS↔IFI	Reject H0
IFI≠ZS	14.3706 ***	4.3273 **	IFI↔ZS	Reject H0

Source: Computed based on Stata 14 result.

^*^ *p*< 0.1.

^**^ *p*< 0.05.

^***^ *p*< 0.01.

**4.2.1 Limitations and future research directions**

This study acknowledges the following limitations. First, it does not account for regional disparities within Ethiopia, which may be shaped by the diverse economic and social landscapes across the country’s regions. Future investigations should prioritize a more detailed analysis that incorporates regional policy perspectives, as this would yield richer insights and support the creation of customized interventions aimed at promoting FI both at the regional and national levels. Secondly, although there were attempts to include digital financial indicators, the lack of transaction data from pertinent authorities hindered the incorporation of these variables into the construction of the index. Consequently, future studies should address this gap. Lastly, upcoming research could gain from the application of alternative data-driven methodologies, such as CFA.

## 5. Conclusion and policy implication

Financial inclusiveness has increasingly become a focal point for policymakers, practitioners, and economists due to its potential to drive inclusive growth. Despite numerous efforts by policy makers and international financial institutions to bolster bank stability and inclusivity, the relationship between FI and bank stability remains contentious and inconsistent in existing literature. This study addresses this gap by examining the impact of FI on bank stability within the Ethiopian context. Using panel data from seventeen commercial banks over the period 2015-2023, the researchers employed a two-stage PCA to develop FI index based on ten Convensional and five digital indicators. The effects of FI on bank stability were analyzed using a two-step robust System GMM approach, complemented by Granger causality tests to determine the direction of causality between FI and bank stability.

Our results from the two-stage PCA indicate that Convensional availability is the most important factor in FI score, followed by Convensional usage, with digital accessibility and digital availability ranking third and fourth, respectively. The System GMM analysis confirms that FI has a significant and positive effect on bank stability. Control variables such as efficiency and GDP growth rate are positively associated with bank stability, while total assets and income diversification exhibit negative effects. Additionally, the historical level of bank stability positively effects current stability. The Granger causality tests indicate a bi-directional relationship between FI and bank stability, suggesting that improvements in FI contribute to enhanced stability, and vice versa.

The policy implications are profound. To promote both FI and stability, policymakers should focus on expanding demographic and geographic outreach, enhancing both convensional and digital financial services. Ensuring that current stability supports future stability is also crucial. FI and bank stability are mutually reinforcing; efforts to enhance FI directly contribute to improved stability and vice versa. Consistent with the policy implication of Vo et al. (2020), broadening FI is essential for strengthening banking sector stability in Ethiopia. Expanding financial services to underserved populations not only provides banks with stable funding sources but also enhances overall sector stability and profitability. Additionally, echoing Oanh et al. (2023) implications, governments should address financial exclusion by removing barriers to access, improving financial education, and developing infrastructure in underserved areas. Policymakers must also ensure that initiatives to promote FI are supported by appropriate macroeconomic policies to sustain the positive relationship between FI and bank stability.

## Ethics and consent

Ethical approval and consent were not required.

## Data Availability

Figshare: Dataset for financial inclusion and stability in Ethiopia case, https://doi.org/10.6084/m9.figshare.27327804.v2 ( Arebo et al., 2024). Data are available under the terms of the Creative Commons Attribution 4.0 International license (CC-BY 4.0).
