# Financial inclusion and stability in Ethiopia using bank-level data: A two-step system GMM estimation

**DOI:** 10.12688/f1000research.158461.2

**Published:** 2025-04-15

**Authors:** Mohammed Arebo, Filmon Hando, Andualem Mekonnen

**Affiliations:** 1 Regional and Local Development Studies, Addis Ababa University College of Development Studies, Addis Ababa, Ethiopia

**Keywords:** Financial Inclusion; Principal Component Analysis; Bank stability; GMM; Ethiopia

## Abstract

**Background:**

This paper examines the impact of FI on bank stability within Ethiopian context, using panel data from 17 commercial banks over the period 2015-2023. Given the scarcity of research focused on the relationship between FI and bank stability in Ethiopia, this paper seeks to address a crucial gap by analyzing both conventional and digital aspects of FI in relation with bank stability.

**Methods:**

A two-stage principal component analysis (PCA) was conducted to construct a composite FI index, integrating 10 conventional and 5 digital indicators. The study applied a two-step robust system generalized method of moments (GMM) to analyze the effects of FI on bank stability, tests nonlinearities using Lind and Mehlum’s (2010) U-test, and examines causality through Dumitrescu-Hurlin (2012) and Juodis et al. (2021) causality tests.

**Results:**

The result reveals an inverted U-shaped relationship between FI and bank stability. FI enhances stability up to a 30.3% threshold, beyond which increased transaction costs, information asymmetries, and adverse selection risks weaken stability. Capital adequacy moderates this effect, raising the threshold to 35.1%, but its stabilizing role diminishes at higher levels. Granger causality tests confirm a bidirectional relationship. Additionally, bank efficiency and GDP growth enhance stability, while real interest rates, total assets, and income diversification exert destabilizing effects.

**Conclusions:**

This study makes three key contributions. First, it provides the first empirical analysis of the FI-stability nexus in Ethiopia. Second: (i), it develops a multidimensional FI index; (ii), explores both linear and nonlinear relationships, and (iii) examines macroprudential regulation as a moderating factor. Third, it tests causality, offering policy insights. To enhance stability while mitigating risks, policymakers must balance FI expansion, enforce regulatory frameworks, and implement targeted capital requirements. Regulators should strengthen consumer protection and financial literacy, while banks must optimize outreach, manage credit risk, and ensure prudent asset allocation and liquidity management to sustain financial stability.

## 1. Introduction

In contemporary financial policymaking, the pursuit of financial system stability has increasingly been viewed in alongside with the promotion of financial inclusion (FI) ( Antwi et al., 2024; Arora, 2020). Growing evidence suggests that the degree of FI can siginificantly impact the stability of financial systems ( Damane & Ho, 2024; Antwi et al., 2024; Vo et al., 2020; Kim et al., 2020; Ahamed & Mallick, 2019). Historically, the dominant focus of financial authorities was primarily on preserving financial stability ( Schinasi, 2005). However, over recent decades, FI has risen to prominence as a key policy goal, complementing the objectives of stability, integrity, and consumer protection, largely driven by the initiatives of global regulatory bodies and stakeholders ( Hannig & Jansen, 2010). This shift prompts a critical inquiry into whether financial stability and FI function as substitutes or complements within the broader regulatory framework ( Morgan & Pontines, 2017).

FI is conceptualized as encompassing the availability, access, and usage of financial products and services ( Nguyen, 2021; Pesqué-Cela et al., 2021; Demirgüç-Kunt et al., 2017; Allen et al., 2016; Sarma, 2016; Babajide et al., 2015). Specifically, “availability” denotes the infrastructural capacity of financial institutions, measured by the number of bank branches, registered agents, and ATMs ( Nguyen & Du, 2022; Sarma, 2016). “Access” (or penetration) reflects the extent to which financial services reach society, operationalized as the number of mobile banking, internet banking, and wallet accounts across commercial banks, microfinance institutions ( Ismael & Ali, 2021; Nguyen, 2021). “Usage” captures the actual adoption of financial products, assessed by the total volume of credit and deposits relative to GDP ( Nguyen & Du, 2022; Nguyen, 2021; Sarma, 2016). Conversely, bank stability, refers to the resilience of financial institutions to economic shocks, ensuring the uninterrupted mediation of savings and investments. It is characterized by the maintenance of adequate capital, effective risk management, and sustained public confidence to avert crises such as bank runs and systemic collapses ( Koudalo & Toure, 2023; Oanh et al., 2023; Anatolyevna & Ramilevn, 2013; Schinasi, 2004). The determinants of bank stability can differ significantly across various contexts, requiring tailored policy responses ( Ozili, 2018). Factors influencing bank stability include internal elements such as general ledger (GL) and profit-loss (PL) accounts, as well as external socio-economic and political conditions ( Ha & Nguyen, 2023). A broader conceptualization of financial stability extends beyond individual banks to capture systemic resilience, market volatility, and rare financial crises ( Cihak, 2020). Ha & Nguyen, (2023) and Borio, (2003), emphasized that stability requires both microprudential measures, aimed at reducing individual bank failures, and macroprudential policies to mitigate contagion effects.

Empirical studies on the impact of FI on bank stability in developing countries is scarce ( Chinoda & Kapingura, 2023; Koudalo & Toure, 2023; Jungo et al., 2022). Neglecting this relationship could lead to severe consequences, including costly financial crises or ongoing financial exclusion ( Danisman & Tarazi, 2020; Čihák et al., 2016). Schoenmaker (1996) highlights that failures within individual financial sectors can trigger contagion effects, leading to widespread instability stemming from the collapse of financial institutions. In such scenarios, a single institution’s failure can create a ripple effect, eroding investor confidence and prompting a rush to liquidate deposits ( Huynh et al., 2020). Therefore, understanding contagion risk is crucial for comprehending the dynamics of the financial system and its stability ( Huynh et al., 2020). Consequently, policymakers must adeptly balance the trade-offs and synergies between fostering FI and maintaining stability ( Damane & Ho, 2024; Čihák et al., 2016).

While FI is widely recognized as a determinant of financial stability ( Oanh et al., 2023), existing studies present divergent perspectives. A dominant strand argues that FI enhances stability by depositor base, mitigating liquidity risk and fostering economic resilience (e.g., Oanh et al., 2023; Koudalo & Toure, 2023; Malik et al., 2022; Nguyen & Du, 2022; Vo et al., 2020; Pham & Doan, 2020; Banna & Alam, 2021; Ahamed & Mallick, 2019; Le et al., 2019; Neaime & Gaysset, 2018; Morgan & Pontines, 2014), while others caution that extending credit to previously unbanked individuals may increase default rates and destabilize financial institutions (e.g., Umar & Akhtar, 2024; Antwi & Kong, 2023; Ha & Nguyen, 2023; Damrah et al., 2023; Barik & Pradhan, 2021; Feghali et al., 2021). However, three critical gaps identified from the existing literature. First, research disproportionately rely on macro-level indicators, analyzing regions such as Sub-Saharan Africa ( Damane & Ho, 2024; Chinoda & Kapingura, 2023; Jima & Makoni, 2023; Anarfo et al., 2020), emerging economies ( Jungo et al., 2022; Wang & Luo, 2022; Vo et al., 2020; Morgan & Pontines, 2017), ASEAN ( Nguyen & Du, 2022; Sethy & Goyari, 2022), Asia ( Malik et al., 2022; Pham & Doan, 2020; Vo et al., 2020; Le et al., 2019), MENA ( Neaime & Gaysset, 2018), Africa ( Koudalo & Toure, 2023; Kebede et al., 2021), and BRICS ( Talbi & Sebai, 2025; Sebai & Talbi, 2024; Barik & Pradhan, 2021). Despite, studies examining FI’s impact at the micro (bank-level) remain limited (e.g., Umar & Akhtar, 2024; Damrah et al., 2023; Anthony-Orji et al., 2022; Matsebula & Sheefeni, 2022). For instance, Ethiopia, where only 10% of households’ access formal credit ( Lakew & Azadi, 2020) and 99.8% of loans are concentrated in urban areas ( NBE-FSR, 2024), remains conspicuously absent from empirical scrutiny. While existing studies in Ethiopia have investigated financial sector stability ( Filatie & Sharma, 2024; Kebede et al., 2024; Fisseha, 2023; Yitayaw et al., 2023; Dress, 2022; Abdu, 2021; Isayas & McMillan, 2021; Aman, 2019; Meher & Getaneh, 2019; Zelie & Wassie, 2019; Meressa, 2018), the impact of FI on bank stability remains largely unexplored. Second, methodological limitations persist, including (i) prior studies have often relied solely on conventional FI indicators, ignoring its multidimensional nature ( Mapurita & Mayoukou, 2024) or focused exclusively on digital aspects (e.g., Antwi & Kong, 2023; Chinoda & Kapingura, 2023; Banna & Alam, 2021). This narrow focus persists despite evidence that digital and traditional channels interact dynamically to shape stability outcomes ( Anton & Nucu, 2024), resulting in an incomplete understanding of FI. (ii), corroborating Damane & Ho, (2024) claims, most previous studies ignored nonlinear dynamics, and insufficient attention moderating impact of macroprudential regulation on FI-bank stability nexus (see Hua et al., 2023). Third, reliance on linear estimators and inadequate causal inference techniques (e.g., OLS, fixed effects) obscures bidirectional relationships and endogenous feedback loops.

This study advances the FI-stability discourse through three novel contributions. First, it addresses the geographic imbalance by focusing on Ethiopia, a paradigmatic case of high financial exclusion (1% rural bank penetration) coupled with systemic concentration risks (23.5% of loans held by top borrowers). Such extremes provide a unique lens to test FI’s stability implications under conditions of structural fragility. Second, this study pushes the frontier by: (i) constructing a multidimensional FI index via two-stage PCA, integrating 10 traditional and 5 digital indicators; (ii) applying a two-step system GMM estimator to mitigate endogeneity and unobserved heterogeneity; (iii) pioneering the examination of the nonlinear FI-stability relationship in Ethiopia, validated through Lind and Mehlum’s (2010) U-test for greater statistical rigor; and (iv) introducing macroprudential regulation as a moderating variable, an overlooked dimension in low-income settings. Third, this study employs Dumitrescu-Hurlin (2012) and Juodis et al. (2021) causality tests to disentangle bidirectional linkages. Finally, the study offers actionable insights for policymakers, guiding strategies to enhance both FI and financial stability in Ethiopia.

The remainder of the study is structured as: Part 2 reviews the literature; Part 3 details the data sources and econometric methods; Part 4 presents and discusses the empirical results; and Part 5 concludes with key findings and policy implications.

## 2. Literature review

### 2.1 Theoretical literature review

FI, from a macroeconomic perspective, is seen as a catalyst for “creative destruction,” promoting economic growth, reducing inequality, and enhancing financial stability. However, at the micro level, excessive FI can lead to opportunistic behaviors, such as fund misuse and deliberate payment delays, which may undermine financial stability ( Hua et al., 2023). The impacts of FI are neither uniform nor static; they vary across different temporal and spatial contexts. This has led to divergent views among researchers regarding its impact on bank stability, with two divergent schools of thought emerging as the one positing a FI-stability effect and the other suggesting a FI-instability outcome ( Damrah et al., 2023; Moaz, 2022). To that end, the relationship between FI and bank stability has become a central theme in both academic and policy discourse ( Čihák et al., 2016). This study employs a multi-theoretical approach, drawing on asymmetric information theory, financial intermediation theory, systemic risk theory, and portfolio theory. These theoretical underpinnings collectively offer a nuanced understanding of the pathways through which FI can effect bank stability in Ethiopia.

Asymmetric information theory, highlighted by Akerlof (1970), addresses the challenges posed by uneven information distribution in financial markets. In FI, this imbalance is particularly significant as institutions serve previously unbanked populations. Difficulties in assessing creditworthiness can lead to adverse selection, hindering credit risk management and operational efficiency, which may compromise financial stability ( Oanh et al., 2023; Moaz, 2022; Sethy & Goyari, 2022; Barik & Pradhan, 2021; Khan, 2011; Bofondi & Gobbi, 2003). Balancing information asymmetry within FI context can thus strengthen the financial system.

The financial intermediation theory underscores the pivotal role of banks in linking savers with borrowers ( Scholtens & Wensveen, 2003; Diamond, 1984), thereby facilitating FI. By providing liquidity, managing risks, and bridging informational gaps, banks contribute to the efficient allocation of capital and the mitigation of risks, which enhances overall financial stability ( Servigny & Renault, 2004). This theory emphasizes that by expanding financial access, particularly in developing economies where financial exclusion is significant, banks can improve their performance by broadening their customer base, increasing deposits, and fostering a more resilient financial system ( Kim et al., 2018; Diamond, 1984). This phenomenon lessens information asymmetry, thereby lessens market imperfections ( Oanh et al., 2023).

Systemic risk theory, as developed by Minsky (1982) and later refined by Borio (2011), addresses the interconnectedness of financial institutions and the amplification of shocks through mechanisms such as leverage, asset price bubbles, and procyclicality. Borio differentiates systemic risk into temporal and cross-sectional dimensions. Temporal risk involves the dynamic evolution of aggregate risk, while cross-sectional risk pertains to the distribution of risk within the financial system, influenced by common exposures and counterparty risks. Within the context of FI, Khan (2011) suggests that expanding borrower bases can lower lending standards, potentially destabilizing the financial system. Conversely, Hannig and Jansen (2010) argue that FI can enhance stability by diversifying deposit and loan bases, involving segments less susceptible to economic cycles, while Prasad (2010) highlights the positive effects of credit access on employment and economic growth.

Portfolio theory highlights the significance of diversification in mitigating risk ( Markowitz, 1952). In the context of FI, a diversified loan portfolio can potentially reduce the impact of credit losses on individual banks and the overall financial system ( Beck et al., 2013; Wang & Lin, 2021). Adem (2022) concurs, noting that diversification lowers bankruptcy risk for banks. Khan (2011) outlines three main ways of mitigating the FI on financialinstability: diversification of bank assets through increased lending to smaller firms reduces portfolio risk; an expanded and stable deposit base lessens reliance on volatile non-core financing; and improved monetary policy transmission enhances stability. However, as Huynh & Dang (2021) argue, the effectiveness of diversification hinges on the correlation between loan segments and the portfolio’s overall risk profile.

**Figure 1.**
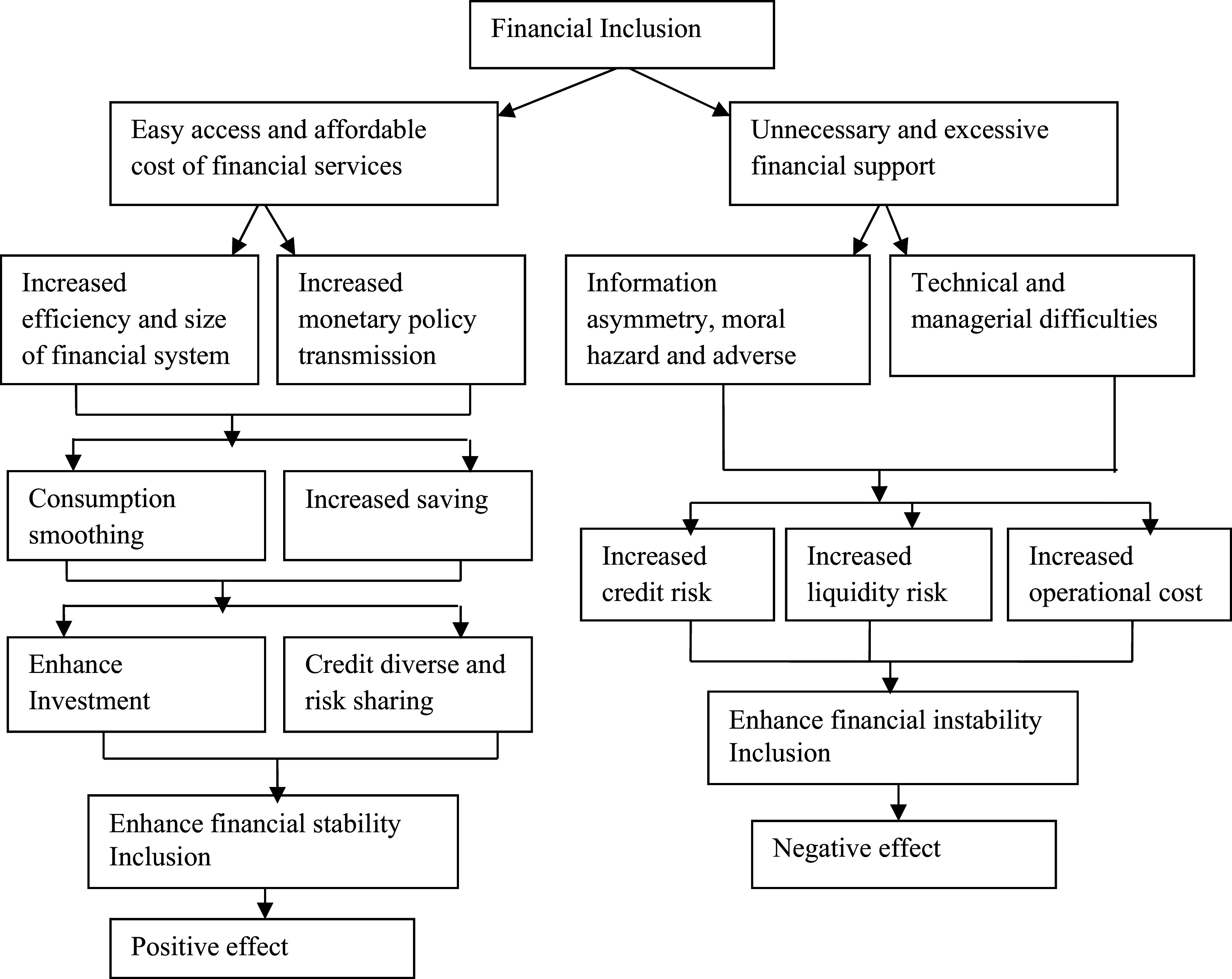
Theoretical channels of interactions between FI and bank stability. Source: Adopted from Damane and Ho (2024), Sethy and Goyari (2022) and Barik and Pradhan (2021).

Drawing on the above theoretical foundations, this study offers a unique lens through which the impact of FI on bank stability can be comprehended. Figure 1 synthesizes these theoretical frameworks, underscoring both stabilizing and destabilizing mechanisms.

On the positive side, corroborating Diamond’s (1984) financial intermediation theory, Oanh (2024) assert that increased intermediation enhances the flow of funds between borrowers and savers, effectively bridging the gap between those with surplus funds and those in deficit. This formalization strengthens monetary policy transmission, optimizes resource allocation, and reduces dependence on informal financial systems ( Damane & Ho, 2024; Sethy & Goyari, 2022). Additionally, FI facilitates consumption smoothing and savings mobilization, expanding banks’ deposit bases and liquidity buffers ( Sethy & Goyari, 2022; Khan, 2011). In accordance with Markowitz’s (1952) portfolio theory, Khan (2011) emphasizes that higher savings lead to increased investment financing and diversified credit access, mitigating loan concentration risk and enhancing risk-sharing mechanisms. Lending to small and medium-sized enterprises (SMEs) further mitigates exposure to single borrowers, lowering the likelihood of non-performing loans (NPLs) and reinforcing stability ( Damane & Ho, 2024). However, excessive FI without proper risk management can undermine financial stability ( Dienillah et al., 2018). Supporting the negative implications of Akerlof’s (1970) information asymmetry theory, ( Damane & Ho, 2024; Sethy & Goyari, 2022), suggests that extending financial services to low-income segments increases adverse selection and moral hazard, elevating credit risk and operational inefficiencies. Specifically, rapid FI expansion often necessitates outsourcing credit assessments and Know Your Customer (KYC) functions, heightening reputational risks and weakening due diligence ( Barik & Pradhan, 2021; Khan, 2011). Furthermore, aligning with Minsky’s (1982) systemic risk theory, Sarmiento (2024), highlights that financial instability within non-bank financial institutions (NBFIs) can spill over into the broader financial ecosystem, amplifies systemic vulnerabilities.

### 2.2 Empirical review and hypothesis development

**2.2.1 Financial inclusion and bank stability**

Empirical research regarding the impact of FI on the stability of financial systems presents varied outcomes ( Damrah et al., 2023; Feghali et al., 2021; Vo et al., 2020). Despite data limitations, these studies provide perspectives on both the positive and negative effects of FI on the stability of banks.

Khan (2011) identified three key channels through which financial inclusion enhances bank stability. First, by promoting asset diversification, FI reduces the risk and cost associated with bank financing, thereby lowering custodial expenses and potential losses. Second, the inclusion of small depositors attracts retail mobilized capital, which is generally more stable than wholesale funding sources. Third, formalization of the informal segment of the population improves the monetary policy transmission, ultimately strengthening the overall stability of the banking system. Wang and Luo (2022), using data from over 1500 commercial banks across 36 emerging economies, found that FI significantly enhances bank stability by promoting operational efficiency, risk management, and funding stability. Similarly, Nguyen and Du (2022), employing System GMM for 102 banks in six ASEAN countries, concluded that FI promotes stability through increased customer deposits and reduced non-performing loans (NPLs). Their findings align with those of Morgan and Pontines (2017), who showed that lending to small and medium sized enterprises (SMEs) diversifies risk and contributes to higher Z-scores and lower NPL ratios, key indicators of bank stability. These studies collectively highlight how extending credit to SMEs serves as a buffer against financial instability by diversifying loan portfolios and reducing concentrated risk exposure.

During financial crises, resources mobilized from FI initiatives are vital for helping institutions escape crisis and enhance resilience ( Ha & Nguyen, 2023; Hannig & Jansen, 2010). Vo et al. (2020) and Chinoda and Kapingura (2023) both underscore the importance of FI in reducing systemic risks. Vo et al. (2020) highlight its role in broadening the depositor base and improving financial resilience in emerging markets, while Chinoda and Kapingura (2023) emphasize the stabilizing effects of digital FI in Sub-Saharan Africa, noting that it significantly reduces NPLs and enhances bank stability. Another layer of analysis emerges in the work of López and Winkler (2019), who explore FI during periods of financial crises using 189 economies data for the period of 2004–2017. Their study finds that countries with higher levels of FI experience less severe disruptions in credit availability during economic downturns. Similarly, Pham and Doan (2020), examining 42 countries across Asia using Feasible Generalized Least Squares (FGLS), conclude that even a weak positive impact of FI on stability is enough to safeguard financial markets against extreme volatility, especially when FI fosters a stable retail deposit base. Jungo et al. (2022) further emphasize the importance of expanding financial access and encouraging investment in FI to maintain monetary system stability, particularly in developing countries. In general, studies like ( Chinoda & Kapingura, 2023; Ha & Nguyen, 2023; Jima & Makoni, 2023; Koudalo & Toure, 2023; Nguyen & Du 2022; Wang & Luo, 2022; Pham & Doan, 2020; Vo et al., 2020; Ahamed & Mallick 2019; Le et al., 2019; Dienillah et al., 2018; Neaime & Gaysset, 2018; Morgan & Pontines, 2014; Hannig & Jansen, 2010), posits that FI contributes to bank stability by expanding the deposit base, diversifying risks, reducing income inequality, and improving regulatory efficiency through policy transmission.

On the other hand, FI has an adverse impact on bank stability. Oanh et al. (2023), claims that FI brings many new, inexperienced clients into the formal financial sector, creating challenges in the credit market. Due to asymmetric information, lenders struggle to assess borrowers’ creditworthiness, with no credit record leads risking stability ( Jima & Makoni, 2023). Damrah et al. (2023) present a nuanced view of FI’s impact on bank stability in Kuwait from 2003 to 2017, illustrating how inclusion can have both positive and negative effects depending on the type of bank (Islamic vs. conventional) and external factors such as financial crises. Their Linear Mixed Model (LMM) reveals that while FI improves access to services, it also introduces inefficiencies and increased risk exposure, particularly during times of economic distress. Their findings align with those of Barik and Pradhan (2021), who, using system-GMM estimators for BRICS nations from 2005 to 2015, argue that rapid credit expansion facilitated by FI can deteriorate lending standards and escalate non-performing loans (NPLs). Employing System GMM ranging from 2011-2019, Umar and Akhtar (2024), examining Chinese banks, found that while FI generally reduces bank risk-taking, it can increase risks in unlisted and large banks, suggesting that the impact of FI varies depending on bank characteristics. Similarly, Le et al. (2019), using Feasible Generalized Least Squares (FGLS), highlight the trade-offs associated with FI, noting that while FI promotes financial sustainability, it also negatively impacts financial efficiency due to the operational challenges of servicing previously unbanked populations. In light of the above empirical literature reviewed, this study proposes the following hypothesis:

H1: Financial inclusion has a positive impact on bank stability

**2.2.2 Non-linear relationship between financial inclusion and bank stability**

A significant limitation of existing empirical research is its predominant focus on the mean effects of FI on financial stability, often overlooking the variation in this relationship across different levels of stability ( Damane & Ho, 2024). Despite the mean impact of FI on bank stability, the findings of ( Sebai et al., 2025; Hua et al., 2023; Khémiri et al., 2024; Ahamed & Mallick, 2019), reveals an inverted U-shaped relationship between FI and financial stability, demonstrating that beyond a certain threshold, further inclusion can destabilize the financial system. Chinoda and Kapingura (2024) identify an inflection point in fintech-driven FI, where early-stage adoption reduces risk-taking but excessive competition later elevates funding costs and operational risks. Contrasting perspectives, such as the U-shaped hypothesis ( Talbi & Sebai, 2025; Antwi et al., 2024; Sebai & Talbi, 2024; Dang & Thi, 2022; Saha & Dutta, 2021), argue that FI’s stabilizing effects resurge at higher development stages.

The above contrasting empirical evidence suggests that FI does not exert a uniform influence on financial stability ( Sebai & Talbi, 2024). Instead, its effects depend on contextual factors such as market concentration, financial sector maturity and institutional quality. Kebede et al. (2021) further emphasize the intricate nature of FI in Africa, noting that its benefits may be limited by high market concentration, which can stifle competition and reduce the effectiveness of inclusion efforts. Dienillah et al. (2018), using a Tobit model for 19 countries from 2004 to 2014, revealed that FI positively impacts stability in high-income nations but shows insignificant effects in lower-income groups. This highlights the need for tailored FI strategies suited to specific economic and regulatory environments. This view is echoed by Oanh et al. (2023) and Jima and Makoni (2023), who highlight the contextual nature of FI’s impact. Employing panel vector autoregression (PVAR) model for 58 countries (27 low financial development states and 31 high financial development states) from 2004 to 2020, Oanh et al. (2023) found that FI positively correlates with stability in low-financial-development countries, but this relationship turns negative in more developed financial systems, where excessive credit access may heighten systemic risks. Similarly, Jima and Makoni (2023), using ARDL cointegration and Granger causality tests from 2000 to 2019 for 26 Sub-Saharan African countries, revealed that while FI contributes to both short-term and long-term stability, the relationship is bidirectional and contingent on broader economic and institutional conditions.

Ahamed and Mallick (2019) extended this argument by analyzing a global dataset of 2635 banks in 86 countries. Using two-step system GMM, they posited that FI enhances stability by increasing customer deposits and lowering marginal banking costs, with the effectiveness of these benefits being contingent on institutional quality. They highlight the role of a robust institutional framework in ensuring that FI does not devolve into unchecked risk-taking. In this context, the study implicitly warns against viewing FI initiatives in a silo, devoid of regulatory oversight or institutional support. Similarly, Hannig and Jansen (2010) emphasized the role of robust institutional frameworks in ensuring that FI initiatives do not lead to unchecked risk-taking, a finding also supported by Sethy and Goyari (2022) in their study of South Asian countries, where FI was found to be a key driver of long-term stability.

In general, based on the observed nonlinear impact of inclusive finance on financial stability ( Sebai et al., 2025; Talbi & Sebai, 2025; Antwi et al., 2024; Anton & Nucu, 2024; Kebede et al., 2024; Hua et al., 2023; Khémiri et al., 2024; Sebai & Talbi, 2024), this study hypothesizes:

H2. There is an inverted U-shaped relationship between FI and bank stability

**2.2.3 The moderating effects of macro-prudential regulation**

Contrary to the assumptions of classical economics, market failure theory underscores the limitations of market forces in efficiently governing the real economy. Risks stemming from information asymmetries and externalities can propagate systemic vulnerabilities, disproportionately affecting traditional commercial banks ( Chinoda & Kapingura, 2024; Pantielieieva et al., 2020). To mitigate such risks, financial regulation emerges as a critical safeguard, curbing excessive risk-taking by banks ( Chinoda & Kapingura, 2024). Among regulatory tools, the capital adequacy ratio (CAR) is a cornerstone of prudential oversight, directly enhancing financial stability by ensuring banks maintain sufficient capital buffers ( Anarfo et al., 2020). While CAR independently strengthens financial stability, its interaction with FI presents another dimension. FI, concieved as expanding access to formal financial services, promotes financial deepening and reduces reliance on informal channels ( Talbi & Sebai, 2025). Yet stringent CAR requirements may inadvertently restrict banks’ lending capacity, resulting in credit rationing that stifles FI ( Anarfo et al., 2020; Gambacorta & Mistrulli, 2004). The stability implications of this interaction hinge on regulatory and institutional contexts. In weak regulatory environments, rapid FI expansion may incentivize reckless risk-taking, eroding the stabilizing effects of capital buffers ( Beck et al., 2013). Conversely, well-capitalized banks are better positioned to manage credit quality, thereby mitigating risks associated with financial deepening ( Thakor, 2014). Robust capital frameworks, when paired with FI, can enhance stability by diversifying revenue streams and reducing dependence on volatile funding ( Anarfo et al., 2020; Hua et al., 2023). However, unchecked credit growth may heighten financial fragility ( Morgan & Pontines, 2014). Since existing literature has not thoroughly explored this interaction, but ( Anarfo et al., 2020; Hua et al., 2023) suggest that the relationship between CAR and FI positively influences bank stability. Owing prior studies, this interaction suggests that FI, when moderated by capital adequacy, strengthens bank stability, leading to the hypothesis:

H3: The interaction between capital adequacy and FI improves bank stability.

**2.2.4 Stylized facts about Ethiopian banking sector**

The empirical literature on FI and financial stability offers a rich diverse of findings, suggesting that a recurring theme across both streams of research is the need to avoid viewing FI or stability in a silo. This concise review of the literature is crucial for our empirical analysis. This is particularly relevant for Ethiopia, where the banking sector exhibits unique structural characteristics that shape the FI-stability nexus. Ethiopian banking sector is state-dominated, with limited competition and underdeveloped capital markets ( Mengesha & Berde, 2023). Government securities are absorbed by captive investors at negative real rates, and the absence of market-based debt issuance reflects systemic inefficiencies ( Abdu, 2022). State-owned Commercial Bank of Ethiopia (CBE) holds 49.5% of total assets and 48.7% of deposits, while five mid-sized banks control 28.0% and 29.4%, respectively, and 24 small banks account for the rest ( NBE-FSR, 2024). Financial deepening is limited, with loans and bonds at 21.4% of GDP and deposits at 24.8% in 2023. Negative real deposit interest rates, driven by high inflation (29.3%), alongside 8% savings deposit rate and a 14.25% lending rate (( NBE Annual Report, 2024), highlight persistent financial distortions. FI remains highly uneven, with less than 1% of loans disbursed to rural areas, while large borrowers (those with credit exposure above 10 million birr) constitute only 0.5% of clients but control 74.8% of total banking sector loans ( NBE-FSR, 2024b). Recent FI strategic reforms, including introduction of interest-free banking, Safaricom Ethiopia’s licensing under the National Digital Payment Strategy (NDPS), and the Digital ID initiative, have increased FI from 10.89% in 2015 to 52.18% in 2023 ( Arebo et al., 2024). Yet, whether these advances enhance stability remains uncertain. The CBE’s dominance creates a bifurcated system as state banks prioritize stability via sovereign debt, while private banks, constrained by thin margins and regulatory asymmetries, limit FI expansion. Negative real returns undermine savings mobilization, while rural exclusion and speculative urban lending lower the threshold at which FI shifts from stabilizing to destabilizing. Hence, this study employs bank-level data to offer appropriately tailored policy implication aimed at enhancing bank stability and FI in Ethiopia.

## 3. Methods

### 3.1 Source and sample of data

Commercial banking institutions in Ethiopia are the most dominant and primary point of access to essential financial services. Based on the data compiled from NBE financial stability report (2024), commercial banks hold for 91.2% of total assets, 97.8% of deposits, 93.9% of credit, and 76.1% of equity in the financial sector, as of June 30, 2023. In light of this dominance, the study focuses on commercial banks but due to data availability concern and the newness of other banks, the researchers focuses on 17 commercial banks. Data for the study sourced from various reliable institutions, like National Bank of Ethiopia (NBE), Commercial banks (CBs) annual report, and World Bank (WB) covering the period 2015-2023.

### 3.2 Construction and identification of variables

**3.2.1 Dependent variables**

Different studies used different indicators to measure bank stability ( Čihák et al., 2016; Damane & Ho, 2024). In this research, Z-score is used as a measure of bank stability, following established literature (e.g., Damrah et al., 2023; Koudalo & Toure, 2023; Barik & Pradhan, 2021; Sethy & Goyari, 2022; Vo et al., 2020; Ahamed & Mallick, 2019). While the Z-score may emphasize profitability, it remains a widely used proxy for bank distance-to-default due to its comprehensiveness, simplicity, and data availability ( Ha & Nguyen, 2023). Due to constraints in risk-related data, this study focuses exclusively on the Z-score. Z-score is valued for its ability to indicate bank insolvency risk, measured as the inverse of the probability of insolvency, reflecting the likelihood that a bank’s assets may not cover its liabilities ( Koudalo & Toure, 2023). Higher returns on assets and greater capitalization improve stability, while lower figures suggest increased risk ( Damrah et al., 2023). This score, calculated from return on assets, volatility, and leverage ( Damrah et al., 2023; Vo et al., 2020), is constructed in this study as follows:

$$Z-{\text{score}}_{\mathrm{it}}=\frac{{\mathrm{ROA}}_{\mathrm{it}}+{\mathrm{EQA}}_{\mathrm{it}}}{\mathrm{sd}{\left(\mathrm{ROA}\right)}_{\mathrm{it}}}$$

Where: **ROA** represents the return on assets, **EQA** denotes the ratio between bank’s total equity and total assets and **Sd** stands with standard deviation.

**3.2.2 Independent variables**

Measuring FI is essential to support evidence-based policy decisions ( Sarma, 2016; Nguyen, 2021). Accurate measurement reveals gaps and opportunities for expanding FI ( Demirgüç-Kunt et al., 2020). However, relying on a single factor can be misleading ( Sarma, 2008; Nguyen, 2021). Composite indices offer a more comprehensive view by integrating multiple dimensions, enabling better comparisons across time and regions ( OECD, 2008). These indices capture the multifaceted nature of FI (Mishra, 2007). This study constructs a multidimensional FI index using two-stage PCA, combining10 convensional indicators and 5 digital indicators (see Table 1).

**Table 1.** Overview of indicators and data sources used in the study.

Variables	Notation	Definition	Sources	Studies
***Bank stability (Dependent variable)***				
Bank Z-Score	ZS	Computes the buffer of a state’s banking system with the volatility of those returns.	NBE	Damrah et al., 2023; Koudalo & Toure, 2023; Barik & Pradhan, 2021; Sethy & Goyari, 2022; Vo et al., 2020; Ahamed & Mallick, 2019
***Financial inclusion indicators (Independent variable**)*				
Convensional Availability	BPC	No of Branches (per 100,000 adults)	NBE; WB	Damrah et al., 2023; Hua et al., 2023; Gharbi & Kammoun, 2023; Jima & Makoni, 2023; Jungo et al., 2022; Barik & Pradhan, 2021; Ismael & Ali, 2021; Kebede et al., 2021; Nguyen, 2021; Sha’ban et al., 2020; Vo et al., 2020; Le et al., 2019; Ahamed & Mallick, 2019; Camara & Tuesta, 2017
Convensional Availability	BAC	No of Branches (per 1000 km ^2^)	NBE; WB	
Convensional Availability	APC	No of ATMs (per 100,000 adults)	NBE; WB	
Convensional Availability	AAC	No of ATMs (per 1000 km ^2^)	NBE; WB	
Convensional Availability	PPC	No of PoSs (per 100,000 adults)	NBE; WB	
Convensional Availability	PAC	No of PoSs (per 1000 km ^2^)	NBE; WB	
Digital Availability	APD	No of agents (per 1,000 adults)	NBE; WB	
Digital Availability	AAD	No of agents (per 1,000 km ^2^)	NBE; WB	
Digital Accessibility	MPD	No of mobile active users (per 1,000 adults)	NBE; WB	
Digital Accessibility	IPD	No of internet active users (per 1,000 adults)	NBE; WB	
Digital Accessibility	WPD	No of mobile money (wallet) users (per 1,000 adults)	NBE; WB	
Convensional Usage	DPC	Depositors with banks (per 1,000 adults)	CBE; WB	
Convensional Usage	APC	No of debit cards per 1,000 adults.	NBE; WB	
Convensional Usage	LGDPC	Outstanding loans and advances (% of GDP)	NBE	
Convensional Usage	DGDPC	Outstanding deposits (% of GDP)	NBE	
***Control variables***				
Loan to Deposit ratio	LDR	Indicates how much of the bank's deposit base is being used for lending.	NBE	Muhammed et al., 2024; Kulu & Bondzie, 2024; Bod’a & Zimková, 2021; Hakim, 2017
Provision to Loan	PL	The loan loss provision ratio represents the funds set aside to cover expected credit losses.	NBE	Ha & Nguyen, 2023; Hua et al., 2023; Koudalo & Toure, 2023; Barik & Pradhan, 2021; Ahamed & Mallick, 2019; Chen et al., 2019
Natural logarithm of Total Asset	lnTA	Provides a comprehensive measure of the bank's financial strength and capacity, reflecting the aggregate value of its economic resources available for generating revenue and supporting operations.	NBE	Damrah et al., 2023; Jungo et al., 2022; Ramzan et al., 2021; Ahamed & Mallick, 2019; Vo et al., 2020; Ali & Puah, 2018; Beck et al., 2013a; Gupta & Kashiramka, 2020
Capital adequacy ratio	CAR	Assesses a bank's ability to absorb potential losses and sustain operations during financial stress, ensuring it maintains sufficient capital to cover its risks and support continued stability	NBE	Hua et al., 2023; Jungo et al., 2022; Kebede et al., 2021; Anarfo et al., 2020; Vo et al., 2020
Income Diversification	IND	The non-interest income to total income ratio insight into the diversification of a bank's income streams beyond traditional interest-based revenue.	NBE	Ahamed & Mallick, 2019; Sanya & Wolfe, 2011; Elsas et al., 2010
Operational efficiency management	EF	It refers to the practices and strategies employed to optimize a bank's operational processes, reduce costs, and enhance productivity.	NBE	Damrah et al., 2023; Ahamed & Mallick, 2019; Beck et al., 2013; Shahriar et al., 2022
Real lending interest rate	RLIR	Inflation adjusted lending interest rate, i.e., lending interest rate minus inflation rate	NBE	Koudalo & Toure, 2023; Anthony-Orji et al., 2022; Atellu & Muriu, 2022; Siddik & Kabiraj, 2018
GDP Growth Rate	GDP	Annual percentage change in gross domestic product	NBE	Damrah et al., 2023; Ha & Nguyen, 2023; Koudalo & Toure, 2023; Oanh et al., 2023; Sethy & Goyari, 2022; Vo et al., 2020; Beck et al., 2013

Source: Prepared by authors.

**3.2.3 Control variables**

As outlined in Table 1, the study includes several bank-specific and macroeconomic control variables, including an index for operational efficiency. While prior studies ( Damrah et al., 2023; Jungo et al., 2022; Sha’ban et al., 2020; Beck et al., 2013) relied on overhead cost as efficiency measure, this study tailors efficiency assessment considering Ethiopia’s deposit-driven banking model. Traditional cost-to-income ratios overlook key operational dynamics, prompting the use of Data Envelopment Analysis (DEA) to develop a more comparable efficiency measure. Non-parametric index is used to drive efficiency using an output-oriented Constant Returns to Scale (CRS) through Data Envelopment Analysis (DEA). The choice of CRS is justified by the structure of the Ethiopian banking industry. Ayalew & Xianzhi (2017), highlight that Ethiopian banks operate within a homogeneous regulatory framework with limited competition. Most banks in Ethiopia are subject to similar capital requirements, operational constraints, and government policies, which result in a uniform relationship between inputs and outputs across banks. Despite Rao & Lakew (2012) suggesting that managers in Ethiopian banks have more control over inputs than outputs, most banks prioritize maximizing profits through efficient use of inputs. Thus, assuming out-put oriented CRS provides a realistic framework for comparing efficiency, as banks are expected to scale their operations proportionally regardless of size, making it suitable for long-term efficiency assessments.

In this study, salary and benefits, provisions, general expenses, branches, and deposits are treated as inputs, while net interest income and non-interest income serve as outputs. Using the model by Charnes et al. (1978) under CRS, the efficiency score θ is calculated as the ratio of weighted sum of outputs to inputs.

$$\text{Efficiency}\;\left(\theta \right)=\frac{\sum_{r=1}^{s}u_{r}Y_{r_{j}}}{\sum_{i=1}^{m}v_{i}X_{i_{j}}}\leq 1,j=1,2,\ldots ,n,$$

Where (θ) = efficiency score of bank j , $Y_{r_{j}}$ = amount of output r produced by bank j , $X_{i_{j}}$ = amount of input i used by bank j , $u_{r}$ = weight assigned to output r , $v_{i}$ = weight assigned to input i , s = number of outputs, m = number of inputs, n = number of banks (decision-making units) being evaluated.

Additionally, the analysis includes control variables such as income diversification, capital adequacy ratio, provisions to non-performing loans, loan-to-deposit ratio, and total assets. Macroeconomic variables include the real lending interest rate and GDP growth rate.

### 3.3 Financial inclusion composite index development strategy

PCA is employed to construct an index that effectively reduces data dimensionality while preserving much information ( Tram et al., 2023; Nguyen, 2021; Hair et al., 2019; Sha’ban et al., 2020; Camara & Tuesta, 2017). It is particularly effective for large, multivariate datasets, addressing challenges like multicollinearity, computational inefficiency, and redundancy by creating uncorrelated variables that maximize variance ( Kyriazos & Poga, 2023; Mavungu, 2023; Jolliffe & Cadima, 2016). Compared to alternative methods, PCA offers several advantages. First, despite non-parametric approaches like Sarma (2008) model, which often rely assigning weights on researcher’s experience ( Tram et al., 2023; Camara & Tuesta, 2017), PCA adapts to changes in data structure ( Decancq & Lugo, 2013), avoiding the pre-assignment of weights before data collection ( Chen et al., 2019). Second, while entropy weighting is objective, studies show it is overly sensitive to minor data fluctuations than PCA ( Chen et al., 2023; Wu & Wang, 2022), leading to unstable weights. Additionally, entropy weighting has limited adoption in FI empirical studies, which undermines its comparability. Third, compared to parametric methods like Confirmatory Factor Analysis (CFA), PCA enhances objectivity by not requiring subjective decisions on factor structure ( Tram et al., 2023; Camara & Tuesta, 2017; Ismael & Ali, 2021; Jolliffe & Cadima, 2016; Steiger, 1979), which may differ across time and space.

Normalization is essential for comparing indicators with varying units and ranges. Common methods include ranking, z-score standardization, min-max rescaling, and logarithmic transformation ( Carrino, 2015; Freudenberg, 2003; OECD, 2008). In this study, the min-max ( MMY ) approach is used to normalize indicators to a scale of 0 to 1, where 0 represents exclusion and 1 represents inclusion.

$$\mathrm{MMY}=\frac{Y_{i}-Y_{\min}}{Y_{\max}-Y_{\min}},$$

Where $Y_{\min}$ , minimum value, $Y_{\max}$ , maximum value.

Traditional single-level multivariate analysis often fails to address nested data structures due to its assumption of independent and identically distributed (i.i.d) observations, potentially missing within group information ( Yang et al., 2022). To mitigate this, a multi-level framework is employed, which addresses the hierarchical nature of the data, enhancing analytical precision ( Yang et al., 2022; Hox, 2013). Additionally, single-stage PCA may disproportionately weight indicators with unequal variances. By generating sub-indices within each dimension separately before combining them, the multilevel approach provides a more balanced and accurate composite index ( Ismael & Ali, 2021; Camara & Tuesta, 2017; Nagar & Basu, 2002).

**3.3.1 First-stage analysis**

Table 2 shows the results of the first-stage PCA. To ensure the suitability of PCA, the Kaiser–Meyer–Olkin (KMO) measure and Bartlett’s test of sphericity are performed. These tests confirm that the sample size is adequate and the indicators are sufficiently intercorrelated ( Ismael & Ali, 2021; Taherdoost et al., 2014). A KMO value of 0.5 or higher and a statistically significant chi-square in Bartlett’s test (1950) indicate that factorization is appropriate ( Hair et al., 2019). As reported in Table 2, all components meet these criteria (KMO ≥ 0.5 and p-value < 0.05), supporting the use of PCA for FI index development. Additionally, internal consistency was measured using a reliability test. According to Kline (2015), a Cronbach’s alpha value above 0.6 is acceptable for developing scales. The obtained Cronbach’s alpha of 0.9721, as shown in Table 2, exceeds this threshold, indicating excellent internal consistency and enhancing the reliability of the data.

**Table 2.** First stage PCA result.

Estimation of PC and eigenvalue of sub-indices of TFI and DFI			
Component	Eigenvalue	Difference	Explained variance
Availability TFI			
Comp1	5.4682	5.1268	0.9114
Comp2	0.3415	0.1566	0.0569
Comp3	0.1849	0.1801	0.0308
Comp4	0.0048	0.0043	0.0008
Comp5	0.0004	0.0002	0.0001
Comp6	0.0003		0
Availability DFI			
Comp1	1.9997	1.9994	0.9999
Comp2	0.0003		0.0001
Accessibility DFI			
Comp1	2.0686	1.2765	0.6895
Comp2	0.7921	0.6528	0.264
Comp3	0.1373		0.0464
Usage DFI			
Comp1	3.4725	2.992	0.8681
Comp2	0.4804	0.443	0.1201
Comp3	0.0374	0.0278	0.0094
Comp4	0.0096		0.0024

Scoring coefficients for orthogonal varimax rotation (weights) and test results							
Dimensions	Indicators	Comp1	Unexplained variance	Normalized weights	KMO	Sphericity test	Cronbach alpha
Convensional availability	BP	0.4146	0.06	0.16927	0.6974	*χ* ^2^(15) = 3347.798 (P = 0.000)	0.9721
	BA	0.4105	0.0786	0.16759	0.6875		
	AP	0.4112	0.0753	0.1679	0.6929		
	AA	0.4078	0.0908	0.16649	0.6851		
	PP	0.3983	0.1324	0.16263	0.6931		
	PA	0.4069	0.0948	0.16612	0.7104		
Digital availability	AP	0.7071	0.0001	0.5	0.5	*χ* ^2^(1) = 1,119.314 (P=0.000)	
	AA	0.7071	0.0001	0.5	0.5		
Digital Accessibility	MP	0.6382	0.1574	0.375736	0.537	*χ* ^2^(3) = 221.805 (P = 0.000)	
	IP	0.4067	0.6578	0.239446	0.7997		
	WP	0.6536	0.1162	0.384818	0.5332		
Convensional usage	DP	0.4691	0.236	0.234706	0.5593	*χ* ^2^(6) = 1,111.108 (P = 0.000)	
	AP	0.5195	0.0627	0.259963	0.6725		
	LGDP	0.4978	0.1396	0.249065	0.5795		
	DGDP	0.5121	0.0892	0.256266	0.654		

Source: Computed based on Stata 14 result.

Hair et al. (2019) deem an explained variance (EV) of 60% or higher as acceptable. Table 2 shows that PC1 captures a significant portion of the variance in the availability and usage dimensions (91.1% and 86.8%, respectively), confirming their substantial role in FI index development. For digital inclusion, PC1 explains nearly 99.9% of the variance in availability, indicating its dominance. As well, PC1 explains 69% of the variance in accessibility, highlighting that while both dimensions are important, they contribute differently to the FI index. Based on Table 2 first-stage PCA result, the following result is built for each indicators, The dimensions of “availability”, and “usage” of the convensional sub-index is;

$${\text{CAvailability}}_{\mathrm{it}}=0.1693{\mathrm{BPC}}_{\mathrm{it}}+0.1676{\mathrm{BAC}}_{\mathrm{it}}+0.1679{\mathrm{APC}}_{\mathrm{it}}+0.1665{\mathrm{AAC}}_{\mathrm{it}}+0.1626{\mathrm{PPC}}_{\mathrm{it}}+0.1661{\mathrm{PAC}}_{\mathrm{it}}+\upsilon_{\mathrm{it}}$$

$${\text{CUsageC}}_{\mathrm{it}}=0.2347{\text{DCPC}}_{\mathrm{it}}+0.26{\mathrm{APC}}_{\mathrm{it}}+0.2491{\text{DGDPC}}_{\mathrm{it}}+0.2563{\text{LGDPC}}_{\mathrm{it}}+\varepsilon_{\mathrm{it}}$$

The analysis reveals that branch availability has the highest relative weight compared to ATMs and PoS machines in both demographic and geographic contexts, emphasizing the crucial role of physical branches in Ethiopia. This finding supports Kebede et al. (2021) and highlights the continued importance of physical branches due to factors such as lower financial literacy, which necessitates in-person interactions. Within the usage dimension, deposit accounts (0.26) hold the highest weight, followed by the deposit to GDP ratio (0.2563), loan to GDP ratio (0.2491), and debit card penetration (0.2347). This underscores the current focus on deposit mobilization in Ethiopia, which collectively emphasizes the sector’s drive to encourage savings. The dimensions of “Availability”, and “Accessibility” of the digital sub-index is given as follows:

$$D{\text{AvailabilityD}}_{\mathrm{it}}=0.5{\mathrm{APD}}_{\mathrm{it}}+0.5{\mathrm{AAD}}_{\mathrm{it}}+{\text{€}}_{\mathrm{it}}$$

$${\text{DAccessibilityD}}_{\mathrm{it}}=0.3757{\text{MBPD}}_{\mathrm{it}}+0.2395{\text{IBPD}}_{\mathrm{it}}+0.3848{\mathrm{WPD}}_{\mathrm{it}}+{\text{€}}_{\mathrm{it}}$$

In digital availability, agency banking indicators (APD and AAD) have equal weights of 0.5, reflecting their equal importance. For digital accessibility, mobile money wallets have the highest weight (0.3848), followed by mobile banking penetration at 0.3757 and internet banking penetration at 0.2394. This indicates that mobile money is the preferred digital financial access method for the general population, contrasting with internet banking, which is primarily used by elitecustomers. This finding aligns with the observation that Ethiopian banks focus internet banking services on premium clients.

**3.3.2 Second-stage analysis**

Table 3 confirms that the KMO measure exceeds the recommended threshold of 0.5 ( Hair et al., 2019), and sphericity test is highly significant (p-value < 0.0001). The first principal component accounts for 80.07% of the total variance, indicating that only 19.97% of the variance remains unexplained. This result confirms the adequacy of the PCA extraction, as supported by Hair et al. (2019). Among four components, only one has an eigenvalue greater than 1, which is retained for constructing the FII. The derived FII is expressed as follows:

$$\mathrm{IFI}=0.2625Y_{i}^{a_{t}}+0.2616Y_{i}^{u_{t}}+0.2254Y_{i}^{a_{d}}+0.2505Y_{i}^{p_{d}}+{℮}_{i}$$

**Table 3.** Second-stage PCA result.

Estimation of PC and eigenvalue of sub-indices of TFI and DFI				
Component	Eigenvalue	Difference	Proportion	Cumulative
Comp1	3.20293	2.67426	0.8007	0.8007
Comp2	0.528673	0.281266	0.1322	0.9329
Comp3	0.247407	0.226421	0.0619	0.9948
Comp4	0.0209866		0.0052	1

Scoring coefficients for orthogonal varimax rotation (weights) and test results					
Indicators	Comp1	Unexplained	Normalized weights	KMO	Sphericity test
Availability	0.524	0.1205	0.2625	0.5948	*χ* ^2^(6) = 709.297 (p = 0.000)
Usage	0.5223	0.1263	0.2616	0.6121	
Availability	0.45	0.3515	0.2254	0.6663	
Accessibility	0.5002	0.1987	0.2505	0.6991	

Source: Computed based on Stata 14 result.

This equation indicates that convensional availability (0.2625) has the highest weight, underscoring the critical role of infrastructure development in enhancing FI. This aligns with findings from Nguyen (2021) and Gharbi and Kammoun (2023), emphasizing the need for robust financial infrastructure alongside efforts in financial literacy and user adoption. Usage (0.2616) is the second most significant dimension, reflecting the importance of account usage in FI. Digital accessibility (0.2505) ranks third, highlighting the increasing impact of digital tools. Agency banking, with the lowest weight (0.2254), suggests that while digital solutions are cost-effective, more targeted interventions are required to address the needs of vulnerable populations, including women, rural residents, and those with lower literacy levels.

To verify the strength of the researchers used the overall average of FI of the country and measured the correlation between the saving and real lending interest rate and newly developed FI index. The Pearson correlation results presented in Table 4 denotes p-values of 0.0000 and 0.0002, respectively, represents that the findings are significant at 1% level (0.9617 for saving) and (-0.9391 for real lending interest rate). The direction of the correlations aligns with theoretical expectations. Real lending interest rates are negatively correlated with FI, as high rates make loans more expensive and discourage access, particularly for low-income populations ( Gharbi & Kammoun, 2023; Uddin et al., 2017). Conversely, in relation to deposits, a positive higher FI scores are associated with in-creased savings, likely due to a greater number of households having access to financial institutions and associated saving products ( Gharbi & Kammoun, 2023). These robust correlations provide strong evidence for the construct validity of the newly developed FI index.

**Table 4.** Pearson correlation between FI index, saving and real deposit interest rate.

		FII
FII	Pearson Correlation	1
Saving	Pearson Correlation	0.9617 ***
	Sig. (bilateral)	0.0000
RLIR	Pearson Correlation	-0.9391 ***
	Sig. (bilateral)	0.0002

Source: Computed based on Stata 14 result.

* *p*< 0.05.

** *p*< 0.01.

*** *p*< 0.001.

### 3.4 Model specification

This study adopts an empirical exploration, employing a panel methodology, to delve into the cross-sectional and longitudinal dynamics of evaluating the impact of FI on bank stability —within the period 2015-2023. The empirical model for examining the impact of FI on bank stability, the researchers estimated this dynamic panel model:

$${\mathrm{ZS}}_{\mathrm{it}}=\beta_{0}+\beta_{1}{\mathrm{IFI}}_{\mathrm{it}}+\beta_{2}{\mathrm{LDR}}_{\mathrm{it}}+\beta_{3}{\mathrm{PL}}_{\mathrm{it}}+\beta_{4}{\text{lnTA}}_{\mathrm{it}}+\beta_{5}{\mathrm{CAR}}_{\mathrm{it}}+\beta_{6}{\mathrm{EF}}_{\mathrm{it}}+\beta_{7}{\mathrm{IND}}_{\mathrm{it}}+\beta_{8}{\text{RLIR}}_{\mathrm{it}}+\beta_{9}{\mathrm{GDP}}_{\mathrm{it}}+\varepsilon_{\mathrm{it}}$$

Where, ${\mathrm{ZS}}_{\mathrm{it}}$ is the measure of bank stability, represented by Z-score, ${\mathrm{IFI}}_{\mathrm{it}}$ , the index of FI extracted from two staged PCA, ${\mathrm{LDR}}_{\mathrm{it}}$ , represents the loan to deposit ratio, ${\mathrm{PL}}_{\mathrm{it}}$ , refers to provision to loan ratio, ${\text{lnTA}}_{\mathrm{it}}$ , indicates the natural logarithm of total asset, ${\mathrm{CAR}}_{\mathrm{it}}$ , denotes risk weighted capital adequacy ratio, ${\mathrm{EF}}_{\mathrm{it}}$ , refers to the efficiency ratio extracted from DEA approach, ${\mathrm{IND}}_{\mathrm{it}}$ , refers to non interest income to total income ratio, ${\text{RLIR}}_{\mathrm{it}}$ , represents inflation adjusted lending interest rate, ${\mathrm{GDP}}_{\mathrm{it}}$ , refers to the gross domestic product growth rate, $\varepsilon_{\mathrm{it}}$ , refers error term, $\beta_{0}$ , refers to the constant term,and $\beta_{1\;\text{to}\;9}$ , represents the slope of the independent variables. ***i*** = 1.. N & ***t*** = 1.. T, denotes to cross-section & time, respectively.

**3.4.1 Baseline analysis applying two-step system GMM**

Traditional FE and RE models, while valuable, often struggle to address endogeneity concerns stemming from omitted variables, measurement errors, reverse causality, and time-invariant unobserved heterogeneity in panel data ( Hill et al., 2020; Leszczensky & Wolbring, 2022). These issues are exacerbated by the presence of lagged dependent variables and endogenous regressors, leading to biased and inconsistent estimates ( Leszczensky & Wolbring, 2022; Bellemare et al., 2017). In the context of dynamic panel data, particularly “small T, large N” panels, selecting an appropriate estimation technique is critical for obtaining reliable and efficient results ( Roodman, 2009)

The Arellano and Bond (1991) estimator is particularly effective in addressing dynamic dependent variables and non-strictly exogenous regressors. By differencing the regressors to eliminate fixed effects and applying GMM, it mitigates potential biases ( Roodman, 2009). The system GMM estimator, introduced by Arellano & Bover (1995) and Blundell & Bond (1998), extends this by assuming the first differences of the instruments are uncorrelated with fixed effects, thus incorporating additional instruments and improving estimation efficiency ( Jima & Makoni, 2023; Bun & Windmeijer, 2010). System GMM constructs a two-equation system, original levels and transformed differences, leveraging both levels and differences of the instruments ( Roodman, 2009). This approach effectively addresses dynamic panel bias, which often distorts small-sample properties and yields unstable estimates when varied instrument sets are employed ( Blundell & Bond, 2023; Windmeijer, 2005).

As noted by Morgan and Pontines (2017), system GMM not only addresses endogeneity bias but also remains consistent and efficient in the presence of heteroskedasticity and autocorrelation within individuals. Our results, as presented in Table 6, confirm the existence of heteroskedasticity and autocorrelation within the model. The system GMM approach is particularly effective in addressing serial correlation and unobserved heterogeneity ( Vo et al., 2020; Roodman, 2009). Therefore, employing GMM to overcome these issues aligns with previous empirical studies ( Jima & Makoni, 2023; Vo et al., 2020; Ahamed & Mallick, 2019; Morgan & Pontines, 2017).

Blundell et al. (2001) and Bond et al. (2001) highlight significant improvements when dealing with models that include a lagged dependent variable along with other explanatory variables. To that end, this study refines the previous model into a dynamic form, ensuring more robust and consistent estimations. To test the first hypothesis, the specified System-GMM model is as follows:

$${\mathrm{ZS}}_{\mathrm{it}}=\alpha_{i}+\mathrm{\varphi i}.{\mathrm{ZS}}_{i,t-1}+\sum_{1}^{n}\beta_{\mathrm{it}}X_{\mathrm{it}}+\in_{\mathrm{it}}$$

In this equation, **ZS**_***it***_ represents the dependent variable, **α**_***i***_ is the individual-specific effect, **φ** is the coefficient for the lagged dependent variable, **β**_***it***_ are the coefficients for the explanatory variables **X**_***it***_, and **∈**_***it***_ is the error term.

The System GMM estimator enhances robustness and efficiency by using both difference and level moment conditions to address endogeneity and ensure reliable inference ( Sebki, 2021; Soto, 2009; Presbitero, 2008). To handle heteroscedasticity and serial correlation, a two-step System GMM procedure with a consistent weighting matrix from one-step residuals was used ( Davidson & MacKinnon, 2004). Although two-step GMM estimates can have downward-biased standard errors ( Presbitero, 2008), the small-sample correction by Windmeijer (2005) was applied to improve efficiency, making it preferable over one-step robust GMM ( Roodman, 2006). Consequently, the two-step robust System GMM estimator was thus selected for further analysis. To prevent overfitting, the maximum number of instrument lags was constrained. The AR(2) and Hansen tests were conducted to validate the absence of misspecification and the appropriateness of the instrumental variables in the GMM estimation.

**Figure 2.**
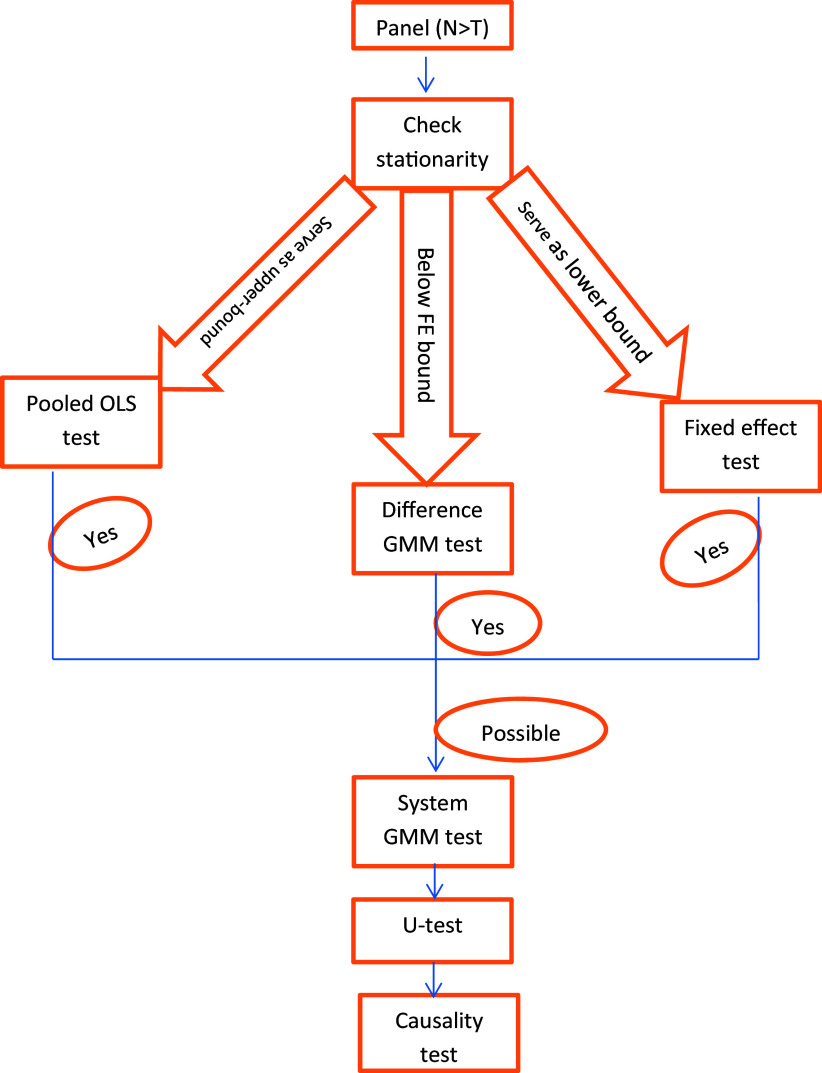
Modeling strategy

The modeling strategy, illustrated in Figure 2, follows a rigorous sequential process to ensure estimator robustness. Initial pooled OLS and fixed effects (FE) estimations establish bounds for the autoregressive parameter (φ), with OLS serving as the upper bound and FE as the lower bound. System GMM is selected over difference GMM when Diff-GMM estimates fall near or below the FE bounds, mitigating weak instrument bias (Azimi, 2022; Bond et al. 2001; Bond, 2002; Presbitero, 2006, 2008). The two-step Sys-GMM estimator is employed with Windmeijer (2005)-corrected standard errors to address heteroskedasticity and serial correlation, enhancing efficiency over one-step alternatives ( Roodman, 2009).

**3.4.2 Non-linearity applying two-step system GMM**

To derive deeper insights, this study examines the potential non-linear relationship between FI and bank stability. A growing body of literature suggests that the FI-stability nexus may exhibit an inverted U-shaped pattern, where moderate levels of financial inclusion enhance stability, but excessive inclusion may introduce risks ( Sebai et al., 2025; Talbi & Sebai, 2025; Khémiri et al., 2024; Sebai & Talbi, 2024; Hua et al., 2023). To empirically test this hypothesis, we adopt a standard quadratic specification of FI’s impact on bank stability:

$${\mathrm{ZS}}_{\mathrm{it}}=\alpha_{i}+\mathrm{\gamma l}.{\mathrm{ZS}}_{i,t-1}+\beta_{1}{\mathrm{IFI}}_{\mathrm{it}}+\beta_{2}{\mathrm{IFI}}_{\mathrm{it}}^{2}+\sum_{1}^{n}\beta_{\mathrm{it}}X_{\mathrm{it}}+\in_{\mathrm{it}}$$

Where, ${\mathrm{ZS}}_{\mathrm{it}}$ **,** denotes bank stability for bank i at a time t , ${\mathrm{ZS}}_{i,t-1}$ , represents one year lag of bank stability, ${\mathrm{IFI}}_{\mathrm{it}}$ , reveals FI score of bank i at a time t **,** ${\mathrm{IFI}}_{\mathrm{it}}^{2}$ **,** captures the non-linear (quadratic) effect of FI, $X_{\mathrm{it}}$ **,** is the vector of control variables, $\alpha_{i}$ **,** is the individual-specific effect, γ **,** is the coefficient for the lagged dependent variable, $\beta_{\mathrm{it}}$ **,** are the coefficients for the explanatory variables $\in_{\mathrm{it}}$ **,** is the error term.

Building on prior research ( Anarfo et al., 2020; Hua et al., 2023), this study further investigates the moderating role of capital adequacy ratio (CAR) in shaping the FI-stability relationship. A well-capitalized banking system may absorb risks associated with financial deepening, mitigating any adverse effects of excessive inclusion. To examine this interaction effect, the baseline model is extended as follows:

$${\mathrm{ZS}}_{\mathrm{it}}=\alpha_{i}+\mathrm{\gamma l}.{\mathrm{ZS}}_{i,t-1}+\beta_{1}{\mathrm{IFI}}_{\mathrm{it}}+\beta_{2}{\mathrm{IFI}}_{\mathrm{it}}^{2}+\beta_{3}{\mathrm{IFI}}_{\mathrm{it}}*{\mathrm{CAR}}_{\mathrm{it}}+\sum_{1}^{n}\beta_{\mathrm{it}}X_{\mathrm{it}}+\in_{\mathrm{it}}$$

Here, ( ${\mathrm{IFI}}_{\mathrm{it}}^{2}*{\mathrm{CAR}}_{\mathrm{it}}$ **)**, captures the interaction between the squared FI term and CAR. A significant coefficient for, $\beta_{3}$ , would indicate that capital adequacy conditions influence the extent to which FI impacts banking stability.

**3.4.2.1 Lind and Mehlum (2010) U-test for non-linearity**

Traditional non-linear models validate an inverted U-shaped or U-shaped relationship by rejecting the null hypothesis when the original and squared terms exhibit opposite signs. However, this approach can be misleading if the true relationship is convex but monotonic, potentially resulting in a false quadratic interpretation ( Law et al., 2017). To address this, Lind and Mehlum (2010) extend a U-test for a U-shaped pattern by evaluating whether the slope is negative at lower values and positive at higher values within the observed range. The U-test verifies these conditions:

$$\beta_{1}+\beta_{2}\left({\mathrm{IFI}}_{l}\right)<0<\beta_{1}+\beta_{2}\left({\mathrm{IFI}}_{m}\right)$$

Where ( ${\mathrm{IFI}}_{l}$ **)**​ and ( ${\mathrm{IFI}}_{m}$ **)**, represent the lower and upper bounds of financial inclusion within the sample. If these conditions hold, the relationship is confirmed as inverted U-shaped.

## 4. Results and discusion

### 4.1 Results

**4.1.1 Descriptive analysis**

Table 5 presents the descriptive summary for the variables used to examine the impact of financial FI on bank stability. The analysis is based on a sample of 153 observations, covering 8 years (2015-2023) across 17 commercial banks (all (17) commercial banks operationalized since 2015 are taken for further analysis). The table provides key details, including the mean, standard deviation, and the minimum and maximum values for each variable.

**Table 5.** Summary analysis.

Variable	Obs	Mean	Std. Dev.	Min	Max
ZS	153	32.403	15.712	8.972	80.117
IFI	153	0.059	0.112	0.002	0.584
LDR	153	0.963	0.089	0.533	1.159
PL	153	0.007	0.010	-0.001	0.087
lnTA	153	10.155	1.320	7.042	14.082
CAR	153	0.188	0.089	0.084	0.958
EF	153	0.893	0.098	0.624	1.000
IND	153	0.284	0.116	0.006	0.570
RLIR	153	-0.036	0.083	-0.196	0.056
GDP	153	0.079	0.015	0.061	0.104

Source: Computed based Stata 14 result.

**4.1.2 Empirical analysis technique**

**4.1.2.1 *Analysis of ordinary least-square regression (OLS)***

Using fixed or random effects models in OLS regression is common for addressing unobserved heterogeneity and simultaneous causality in panel data ( Kumar et al., 2022). Fixed effects handle time-invariant characteristics within firms ( Schultz et al., 2010), while random effects reduce variability by pooling data and accounting for differences across firms and time ( Kumar et al., 2022). The Hausman test, which checks for the correlation between unique errors and regressors, guides the choice between these models ( Kumar et al., 2022; Greene, 2003). Our results indicate that the fixed-effects model is the correct choice, as shown in Table 9.

To test for autocorrelation and heteroskedasticity, the Wooldridge, modified Wald, and Breusch-Pagan (BP) tests were employed. Results in Table 6 reveals, both problems were detected. However, the correlation matrix and Variance Inflation Factor (VIF) results in Table 7, with all VIF values lessthan 10, suggest that multicollinearity is not a concern. Following Vo et al. (2020) and Ahamed and Mallick (2019), robust clustered standard errors were applied to address these issues, as outlined in Table 9.

**Table 6.** Heteroscedasticity and autocorrelation test.

Tests	Wooldridge test	Modified Wald test	Breusch-Pagan (BP) test
Type	Serial correlation	Heteroscedasticity	Heteroscedasticity
	F-test	Chi ^2^ test	Chi ^2^ test
Sig level	*F(1,16)=30.970 (p=0.0000)*	*χ ^2^(17)=112.98 (p=0.0000)*	*χ ^2^(1)=7.54 (p=0.0060)*

Source: Computed based on Stata 14 result.

**Table 7.** Multicollinearity test.

Variables	VIF	1/VIF	ZS	IFI	LDR	PL	TA	CAR	EF	IND	RLIR
IFI	3.94	0.254	-0.29								
LDR	1.43	0.701	-0.02	0.423							
PL	1.12	0.894	-0.35	0.262	0.215						
lnTA	5.69	0.176	-0.16	0.718	0.364	0.220					
CAR	1.26	0.791	-0.1	0.045	-0.125	-0.007	-0.209				
EF	1.36	1.3	0.771	0.203	0.323	0.082	0.202	0.012			
IND	2.41	0.414	-0.07	-0.253	-0.282	-0.076	-0.647	0.417	-0.039		
RLIR	3.03	0.33	0.071	0.000	-0.160	-0.138	-0.462	0.255	-0.308	0.446	
GDP	2.32	0.431	0.071	0.000	-0.170	-0.112	-0.395	0.230	-0.195	0.460	0.736

Source: Computed based on Stata 14 result.

**4.1.3 GMM analysis**

**4.1.3.1 Stationary test**

Conducting a unit root test is crucial to confirm stationarity of variables, thereby avoiding the risk of spurious findings ( Breitung & Pesaran, 2005). Although a dynamic panel approach is typically suited for variables integrated at level or first difference, it is crucial to ensure none of the variables is integrated at a higher order, such as I(2) ( Pesaran & Smith, 1995). To verify stationarity, we employed the Levin-Lin-Chu (LLC) and Harris-Tzavalis (HT) unit root tests across both I(0) and I(1) levels, as shown in Table 8. The LLC test confirms that all variables, are stationary at level. Similarly, the HT test shows that only total assets achieve stationarity at first difference. Since all variables are either stationary at level or first difference, applying the GMM method is appropriately validated.

**Table 8.** Stationary test result: LLC and HT.

	LLC		HT	
Variables	T-statistics	Order	T-statistics	Order
ZS	(7.1564) ***	I(0)	0.5428 *	I(0)
IFI	(6.7882) ***	I(0)	(0.0528) ***	I(0)
LDR	(3.7723) ***	I(0)	0.1663 ***	I(0)
PL	(17.1597) ***	I(0)	0.1221 ***	I(0)
lnTA	(6.7186) ***	I(0)	0.0677 ***	I(1)
CAR	(8.2427) ***	I(0)	(0.0436) ***	I(0)
EF	(2.9716) **	I(0)	0.425 ***	I(0)
IND	(3.8704) ***	I(0)	(0.2079) ***	I(1)
RLIR	(8.5431) ***	I(0)	(0.0852) ***	I(0)
GDP	(5.8930) ***	I(0)	0.2165 ***	I(0)

Source: Computed based on Stata 14 result.

* p < 0.05.

** p < 0.01.

*** p < 0.001.

Following Bond et al.’s (2001) recommendation, the Pooled OLS coefficient served as an upper bound, while the Fixed Effects coefficient acted as a lower bound. Given that the Difference GMM coefficients (0.347 and 0.328) were significantly lower than the Fixed Effects coefficient (0.412) and well below the Pooled OLS coefficient (0.947), as presented in Table 9, the System GMM approach was deemed appropriate.

**Table 9.** Empirical result of the study.

Variables	(1) Robust Fixed Effect	(2) Robust Pooled OLS	(3) Robust One-step Difference GMM	(4) Robust Two-step Difference GMM	(5) Robust One-step System GMM	(6) Robust Two-step System GMM
l.ZS	0.412 ^***^	0.947 ^***^	0.347 ^**^	0.328	0.798 ^*^	1.155 ^***^
	(5.62)	(55.49)	(2.15)	(1.15)	(2.08)	(3.07)
IFI	-8.811	-2.890	-10.41	-9.382	49.93 ^**^	84.07 ^**^
	(-0.99)	(-0.52)	(-1.00)	(-0.65)	(2.85)	(2.60)
LDR	10.82 ^***^	1.010	11.51 ^***^	9.283	-19.10	-55.46
	(3.43)	(0.26)	(2.95)	(1.74)	(-1.07)	(-1.73)
PL	-69.73 ^**^	-63.76 ^**^	-70.40 ^**^	-63.67 ^*^	-125.7	17.66
	(-2.71)	(-2.27)	(-2.77)	(-1.96)	(-0.42)	(0.07)
lnTA	-2.249 ^**^	0.321	-2.342 ^**^	-1.937 ^*^	-8.085 ^***^	-10.03 ^**^
	(-2.30)	(0.65)	(-2.39)	(-1.83)	(-3.04)	(-2.66)
CAR	3.488	0.747	3.566	1.414	10.68	-37.80
	(0.71)	(0.45)	(0.69)	(0.18)	(0.41)	(-0.99)
EF	13.61 ^***^	7.436 ^**^	14.04 ^***^	13.21 ^*^	15.35	26.24 ^*^
	(3.56)	(2.60)	(3.65)	(2.00)	(1.46)	(2.00)
IND	6.825 ^*^	1.230	7.208 ^*^	5.309	-71.81	-101.7 ^*^
	(1.80)	(0.41)	(1.80)	(1.09)	(-1.39)	(-1.94)
RLIR	-11.38	-6.605	-10.55	-6.237	-32.19 ^*^	-33.46
	(-1.54)	(-1.26)	(-1.41)	(-0.64)	(-1.99)	(-1.42)
GDP	9.593	29.75	7.668	4.417	105.3	229.5 ^*^
	(0.42)	(1.05)	(0.35)	(0.19)	(1.38)	(2.12)
_cons	16.42 ^*^	-12.12			99.98 ^**^	138.4 ^**^
	(1.86)	(-1.58)			(2.92)	(2.44)
*N*	136	136	119	119	136	136
*Groups*	17		17	17	17	17
*Instruments*	-	-	16	16	16	16
*AR(2) test*	-	-	-1.46 (0.144)	-1.38 (0.167)	-0.12 (0.908)	-0.71 (0.479)
*Hansen test*	-	-	10.55 (0.103)	10.55 (0.103)	4.12 (0.532)	1.13 (0.951)
*R-Squared *	0.7632	0.9665				
*P-Value *	F(10,16) = 68.14 (p=0.000)	F(10,125) = 413.07 (p=0.000)	F(10,17) = 13.59 (p=0.000)	F(9,143) = 8.49 (p=0.000)	F(10,16) = 2104.9 (p=0.000)	F(10,16) = 592.07 (p=0.000)

Source: Computed based on stata 14 result.

^*^ *p*< 0.1.

^**^ *p*< 0.05.

^***^ *p*< 0.01.

Beyond estimating the mean impact of FI on bank stability using two-step robust system GMM, this study investigates potential non-linearity in this relationship. To rigorously investigate the presence of a non-linear effect, the Lind and Mehlum (2010) U-test is employed, incorporating a quadratic specification to capture possible inverted U-shaped dynamics. Furthermore, the findings presented in Table 10 highlight the moderating role of CAR in the FI–bank stability nexus. Specifically, the results illustrate the non-linearity both with and without the interaction term, providing deeper understanding of FI’s conditional effects on stability.

**Table 10.** Non-linearity test (two step robust system GMM with and with-out interaction term).

Variables	(7) Non-linearity with interaction term		(8) Non-linearity without interaction term	
l.ZS	0.212		0.240	
	(0.72)		(0.79)	
FI	513.5 ***		417.8 **	
	(3.15)		(3.14)	
FI^2	-731.3 **		-688.5 **	
	(-3.20)		(-3.01)	
CAR	20.41		3.213	
	(1.41)		(0.32)	
FI*CAR	-305.9 **			
	(-2.24)			
LDR	14.00		9.522	
	(0.77)		(0.53)	
PL	-380.0 **		-377.0 *	
	(-2.71)		(-2.70)	
LnTA	-12.44 ***		-11.81 **	
	(-3.91)		(-3.58)	
EF	21.86 *		22.11	
	(1.80)		(1.84)	
IND	-44.71 *		-36.93	
	(-1.95)		(-1.74)	
RLIR	-30.23 **		-30.50 *	
	(-2.29)		(-2.31)	
GDP	70.02 *		58.12	
	(2.05)		(1.73)	
_cons	108.3 ***		108.3 **	
	(4.24)		(3.81)	
*N*	136		136	
*Groups*	17		17	
*Instruments*	17		16	
*AR(2) test*	-0.83 (p=0.405)		0.14(p=0.888)	
*Hansen test*	1.32 (p=0.857)		1.91(p=0.752)	
*P-Value*	F(12,16) = 2943.73 (p=0.000)		F(11,16) = 3998.22 (p=0.000)	
**Inverse U-test ( Lind & Mehlum, 2010)**				
*Test components*	Value	Significant	Value	Significant
*Turning point*	IFI = 0.351		IFI = 0.303	
*Slope at lower bound*	510.03	p = 0.003 ***	414.51	p = 0.003 ***
*Slope at upper bound*	-340.63	p = 0.003 ***	-386.3	p = 0.006 ***
*Overall test (t-value)*	3.15	p = 0.003 ***	2.85	p = 0.006 ***

* *p*< 0.1.

** *p*< 0.05.

*** *p*< 0.01.

**4.1.4 Causality test**

This research examines the dynamic causal relationship between FI and bank stability within the Ethiopian banking sector. Both the Dumitrescu and Hurlin (2012) W-statistic test and the Juodis et al. (2021) Z-bar test are used to ascertain the direction of causality between FI and bank stability. Rejection of the null hypothesis indicates a causal relationship, whereas its acceptance suggests none ( Antwi et al., 2024). The analysis employs a first lag, with lag selection based on the Modified Bayesian Information Criterion (MBIC) to ensure result robustness, in line with Hussain et al. (2024) and Žiković et al. (2020) recommendations for shorter time periods.

### 4.2 Discusion

The findings reveal that the lagged value of bank stability positively effect current stability. Specifically, past levels of bank stability significantly impact present stability, as corroborated by the study of Yitayaw et al. (2023) within the Ethiopian context. The positive effect of lagged stability on current conditions underscores its validity as a reliable instrument for stability analysis, given that banks generally exhibit continuity in their stability across different periods, reflecting a trend of persistence over time. However, in column 7 and 8 ( Table 10), where non-linear FI terms ${\mathrm{IFI}}_{\mathrm{it}}^{2}$ and interaction effects ( ${\mathrm{IFI}}_{\mathrm{it}}*{\mathrm{CAR}}_{\mathrm{it}}$ **)** are introduced, the lagged term loses significance. This suggests that stability persistence is conditional on evolving financial dynamics, as non-linearity and regulatory adjustments reshape stability paths, reducing reliance on historical trends.

Our findings confirm a significant positive relationship between FI and bank stability, supported by existing research ( Koudalo & Toure, 2023; Nguyen & Du, 2022; Vo et al., 2020; Le et al., 2019; Neaime & Gaysset, 2018; Morgan & Pontines, 2014). The result clearly supports hypothesis 1. Specifically, a 1% increase in FI is associated with an 84.07 unit increase in bank stability in the short run, ceteris paribus. This outcome can be attributed to several factors. First, enhanced FI strengthens bank stability by diversifying bank assets, reducing systemic and liquidity risks, and improving monetary policy transmission ( Ahamed & Mallick, 2019; Morgan & Pontines, 2014; Khan, 2011; Hannig & Jansen, 2010). It also addresses information asymmetry by allowing lenders to better assess borrowers and by providing banks with critical proprietary information ( Nguyen & Du, 2022), thus fostering a more resilient banking sector. Third, greater FI expands deposit bases, reducing the procyclicality risk of the banking sector ( Chinoda & Kapingura, 2024; Ozili, 2018), further reinforcing financial stability.

Building on prior studies ( Sebai et al., 2025; Talbi & Sebai, 2025; Antwi et al., 2024; Kebede et al., 2024; Khémiri et al., 2024; Sebai & Talbi, 2024; Hua et al., 2023; Dang & Thi, 2022; Saha & Dutta, 2021; Ahamed & Mallick, 2019), our findings in Column 8 confirm an inverted U-shaped relationship between FI and bank stability, supporting Hypothesis 2. This indicates that while FI enhances stability up to a certain threshold, beyond that point, its impact reverses. This result aligns with studies showing a positive effect of FI on bank stability ( Sebai et al., 2025; Chinoda & Kapingura, 2024; Khémiri et al., 2024; Hua et al., 2023) but contrasts with others reporting a destabilizing effect at lower FI levels ( Talbi & Sebai, 2025; Antwi et al., 2024; Sebai & Talbi, 2024; Dang & Thi, 2022; Saha & Dutta, 2021). At lower bounds, FI fosters stability by mitigating asymmetric information, enabling banks to diversify their portfolios ( Chinoda & Kapingura, 2024; Khémiri et al., 2024), and enhancing the quality of bank-client relationships, which reduces risk premiums and promotes financial transparency ( Khémiri et al., 2024). However, at higher levels, in line with Khan (2011) and Khémiri et al., (2024), the mass participation of low-income individuals in formal banking raises transaction and information costs, increasing adverse selection and moral hazard risks. This dynamic can trigger credit contractions and asset quality deterioration, leading to financial distress ( Pham & Doan, 2020). The Lind and Mehlum (2010) U-test results in Column 8 confirm this threshold effect, with the lower bound slope (414.5) exceeding the upper bound (-386.3), indicating that stability gains diminish as FI expands beyond a critical point. Specifically, incorporating a quadratic FI term (IFI ^2^) reveals a stability-maximizing threshold at IFI ≈ 30.3% (U-test, p = 0.006), contrasting with Khémiri et al. (2024), who found destabilization only at much higher FI levels (~71.6%) in GCC economies. These findings highlight the importance of balancing financial inclusion efforts to ensure long-term banking stability, as excessive credit concentration can erode diversification benefits, necessitating prudent regulatory oversight to maintain stability beyond the optimal FI threshold.

The moderating effect of the interaction ( ${\mathrm{IFI}}_{\mathrm{it}}*{\mathrm{CAR}}_{\mathrm{it}}$ ) shifts the inflection point from 0.30 (without interaction) to 0.35 (with interaction), confirming Hypothesis 3, that the interaction between capital adequacy and FI enhances bank stability. This indicates that stronger regulatory frameworks can improve the FI–stability relationship by mitigating financial risks and fostering transaction efficiency ( Ha & Nguyen, 2023). However, beyond the threshold, stability gains diminish, and the negative FI*CAR interaction suggests that higher capital adequacy does not necessarily shield banks from excessive FI risks. This could stem from diminishing capital returns, rising regulatory costs, and increased risk exposure. In Ethiopia, where government intervention influences financial allocation ( Filatie & Sharma, 2024), banks face unique stability risks as political priorities override market-driven risk management. Thus, while capital adequacy enhances stability in financially inclusive environments, its protective effects weaken when FI expands beyond sustainable levels.

Our findings indicate that control variables such as lnTA, EF, IND, RLIR and GDP growth rate significantly affect bank stability. Larger banks exhibit decreased stability, with a 10.03 unit decline per percentage increase in total assets in the short run, ceteris paribus. This effect is evident in both non-linear analysis (see column 7 and 8). This aligns with Mirzaei et al. (2013), who caution that excessive size amplifies systemic vulnerabilities. In Ethiopia’s concentrated banking sector, state-owned CBE dominate ~50% of assets ( NBE-FSR, 2024b), fostering operational rigidity and reliance on implicit government guarantees. The “too-big-to-fail” (TBTF) phenomenon fosters moral hazard, encouraging excessive risk-taking ( Farhi & Tirole, 2012) and increasing instability due to complexities in governance and risk management ( Shahriar et al., 2022; Vo et al., 2020). This finding aligns with Vo et al. (2020) and Mu and Lin (2016), but differing from Nguyen and Du (2022) and Ahamed and Mallick (2019).

Efficiency positively effect stability, with a 1% increase in efficiency leading to a 26.24 unit rise in stability in the short run, ceteris paribus. This effect is the same in both non-linear analysis (see column 7 and 8). Efficient banks optimize resource allocation, strengthen profitability, and enhance risk management ( Gupta & Raman, 2020; Mohapatra & Mohanty, 2017). Empirical evidence links higher efficiency to lower non-performing loans and stronger financial resilience ( Shahriar et al., 2022; Berger & DeYoung, 1997). Efficiency also bolsters market power, liquidity buffers, and long-term sustainability ( Kasman & Carvallo, 2014; Fiordelisi et al., 2011). This finding consistent with Nguyen and Du (2022), and Ahamed and Mallick (2019).

Income diversification negatively impacts stability, with a 1% increase resulting in a 101.7 unit decrease in stability in the short run, ceteris paribus. This effect is particularly evident in model 7 but insignificant in model 8. Over-reliance on non-interest income exposes banks to systemic risks, particularly when diversification extends into unfamiliar sectors ( Carlson, 2004). Excessive diversification into volatile markets such as forex trading amidst chronic dollar shortages heightens asymmetric information risks ( Acharya et al., 2006). Mercieca et al. (2007) emphasize that diversification benefits depend on regulatory quality and market discipline. In the Ethiopian context, where banks are predominantly dependent on deposit revenue, an abrupt or poorly managed shift towards greater non-interest income might contribute to instability. This finding aligns with Nguyen and Du (2022) but differing from Ahamed and Mallick (2019).

GDP growth rate shows a positive effect on stability, with a 1% increase leading to a 229.5 unit rise in stability in the short run, ceteris paribus. This finding remains robust in non-linear analyses with interaction terms but loses significance when interaction effects are excluded. Economic expansion enhances financial stability through multiple channels. A growing economy improves borrower creditworthiness, thereby reducing default risks and strengthening bank balance sheets ( Demirgüç-Kunt & Maksimovic, 1998). Additionally, higher GDP growth fosters employment and business profitability, ensuring that debt obligations are met, thereby reducing non-performing loans and bolstering banking sector stability. Financial deepening associated with economic expansion further strengthens banks’ capital positions and shock-absorbing capacity ( Fosu et al., 2017). This finding consistent with Nguyen and Du (2022), Vo et al. (2020), and Ahamed and Mallick (2019).

The real lending interest rate (RLIR) exhibits a negative impact on bank stability in non-linear specifications, consistent with the findings of Anthony-Orji et al. (2022) and Atellu and Muriu (2022). Prolonged low-interest-rate environments compress net interest margins (NIMs), compelling banks to engage in riskier lending practices to sustain profitability ( Rajan, 2006; Adrian & Shin, 2009). This “search-for-yield” behavior often leads to capital misallocation and elevated exposure to financial shocks. Furthermore, artificially low interest rates contribute to “money illusion,” distorting risk-adjusted returns and weakening credit standards ( López-Penabad et al., 2022; DellʼAriccia et al., 2014). Simultaneously, low rates undermine banks’ ability to build capital buffers through retained earnings, heightening vulnerability to liquidity constraints ( Zimmermann, 2019). The destabilizing effects of low interest rates are particularly pronounced in emerging markets, where financial systems exhibit structural weaknesses and heightened interconnectedness ( Beck et al., 2015; Calice et al., 2020). Interest rate repression through regulatory caps further distorts risk pricing, compelling banks to either ration credit or extend loans to subprime borrowers, thereby inflating non-performing loans ( Chen et al., 2017; Beck et al., 2015). Relatedly, Apergis et al., (2022), highlight that inflationary pressures exacerbate systemic risks, further reinforcing the precarious balance between monetary policy and financial stability.

The two-step System GMM analysis, as detailed in Table 9 and Table 10, shows that the standard diagnostic tests are supportive of the model’s validity. The AR(2) test indicates no evidence of second-order residual autocorrelation, with (Prob > χ ^2^ = 0.479) (model 6), (Prob > χ ^2^ = 0.405) (model 7) and (Prob > χ ^2^ = 0.888) (model 8), thereby validating the absence of misspecification in the dynamic panel model. Similarly, the Hansen test (Prob > χ ^2^ = 0.951) (model 6), (Prob > χ ^2^ = 0.857) (model 7) and (Prob > χ ^2^ = 0.752) (model 8), confirms the validity of the instruments, verifying their appropriateness. The F-test (Prob > F = 0.000) for both linear and non-linear specifications demonstrates a strong goodness of fit for the model. In addition to the two-step robust System GMM results, findings from robust FE, robust Pooled OLS, robust one-step Difference GMM, robust two-step Difference GMM, and robust one-step System GMM are largely consistent. The main variable, FI, is significant in both one-step and two-step System GMM models, while the GDP growth rate is significant in the two-step robust System GMM. Overall, these results underscore the robustness of the employed models.

In addition, based on the result revealed in Table 11, the Juodis et al. (2021) test confirms that FI significantly Granger-causes bank stability at the 5% level, a result supported by the Dumitrescu and Hurlin (2012) test, which also rejects the null hypothesis. This underscores the substantial role of FI in bolstering the resilience and stability of Ethiopia’s banking sector. Additionally, reverse causality from bank stability to FI is significant at the 5% level, indicating that bank stability can predict enhancements in FI. These findings suggest that enhancing FI can improve bank stability, while a stable financial environment fosters greater FI, aligning with previous studies ( Jima & Makoni, 2023; Antwi et al., 2024).

**Table 11.** Causality test result.

Test	Z-bar	P-Value	Direction	Conclusion
ZS≠IFI	7.3252 ***	95.3201 ***	ZS↔IFI	Reject H0
IFI≠ZS	14.3706 ***	4.3273 **	IFI↔ZS	Reject H0

Source: Computed based on Stata 14 result.

* *p*< 0.1.

** *p*< 0.05.

*** *p*< 0.01.

## 5. Conclusion, and policy implication, limitations and future research directions

### 5.1 Conclusion

Financial inclusiveness has increasingly become a focal point for policymakers, practitioners, and economists due to its potential to drive inclusive growth. Despite numerous efforts by policy makers and international financial institutions to bolster bank stability and inclusivity, the relationship between FI and bank stability remains contentious and inconsistent in existing literature. This study addresses this gap by examining the impact of FI on bank stability within the Ethiopian context. Using panel data from seventeen commercial banks over the period 2015-2023, the researchers employed a two-stage PCA to develop FI index based on ten convensional and five digital indicators. The effects of FI on bank stability were analyzed using a two-step robust System GMM approach while testing non-linearities via Lind and Mehlum’s (2010) U-test, complemented by Granger causality tests to determine the direction of causality between FI and bank stability.

Our results from the two-stage PCA indicate that convensional availability is the most important factor in FI score, followed by convensional usage, with digital accessibility and digital availability ranking third and fourth, respectively. The System GMM analysis confirms that FI has a significant and positive effect on bank stability. However, the relationship between FI and stability is not linear. Instead, the study reveals an inverted U-shaped pattern, wherein FI contributes positively to stability up to a threshold of approximately 30.3%, beyond which its effects become destabilizing. This turning point reflects the trade-offs associated with excessive FI, as mass participation in the banking system, particularly among low-income individuals, can heighten transaction costs, exacerbate information asymmetries, and increase the likelihood of adverse selection and moral hazard. The introduction of capital adequacy as a moderating factor extends the stability-maximizing threshold to 35.1%, illustrating that stronger regulatory buffers can mitigate some of the risks associated with higher FI. However, beyond this point, capital adequacy ceases to provide stability benefits, as diminishing returns and heightened regulatory costs erode its effectiveness. Control variables such as efficiency and GDP growth rate are positively associated with bank stability, while real interest rate, total assets and income diversification exhibit negative effects. Additionally, the historical level of bank stability positively effects current stability. The Granger causality tests indicate a bi-directional relationship between FI and bank stability, suggesting that improvements in FI contribute to enhanced stability, and vice versa.

This study makes three contributions to the field. First, it is the first to empirically investigate the effect of FI on banking stability in Ethiopia, addressing a critical knowledge gap in a financial landscape where FI is still in its nascent stages. Second, methodologically, (i), unlike prior studies that have predominantly relied on either traditional or digital FI indicators in isolation, this research develops a multidimensional FI index using a two-stage PCA approach, integrating ten traditional and five digital indicators. (ii), this study applies a two-step robust system GMM estimator to address endogeneity concerns and unobserved heterogeneity, thereby enhancing the reliability of the results. (iii), it pioneers the investigation of nonlinear FI-stability relationship in Ethiopia, validated through Lind and Mehlum’s (2010) U-test, a more robust approach to validating nonlinearity than conventional quadratic models; (iv), this study introduces macroprudential regulation as a moderating factor, a dimension largely overlooked in low-income economies. The findings illustrate that while capital adequacy strengthens stability at moderate FI levels, its mitigating effects diminish beyond a certain threshold, signaling the need for more adaptive regulatory frameworks. Third, in response to concerns regarding reverse causality, the study employs Dumitrescu-Hurlin (2012) and Juodis et al. (2021) Granger causality tests, providing empirical evidence on the directional nexus between FI and stability. Finally, the study extends its impact beyond academic discourse by offering contextual actionable strategies to optimize FI expansion without compromising financial stability.

### 5.2 Policy implication

The policy implications are profound. To promote both FI and stability, policymakers should focus on expanding demographic and geographic outreach, enhancing both convensional and digital financial services. Ensuring that current stability supports future stability is also crucial. FI and bank stability are mutually reinforcing; efforts to enhance FI directly contribute to improved stability and vice versa. Consistent with the policy implication of Vo et al. (2020), broadening FI is essential for strengthening banking sector stability in Ethiopia.

Specifically, the findings of this study suggest several policy implications that should be considered. First, the identified inflection point of 35%, coupled with the moderating effect of capital adequacy, highlights that while policy interventions promoting FI up to this threshold will enhance stability, further expansion requires nuanced regulatory oversight. Banks must recognize that internal buffers such as capital adequacy may not act as perfect hedges against the risks associated with excessive FI. This is particularly critical in Ethiopia, where current FI levels remain below 0.1 for all commercial banks except CBE ( Arebo et al., 2024), suggesting ample room for expansion before the stability-maximizing threshold is reached. Second, expanding financial services to underserved populations not only provides banks with stable funding sources but also enhances overall sector stability and profitability. However, as financial access increases, additional risk mitigation strategies must be implemented. Echoing the recommendations of Oanh et al. (2023), governments should address financial exclusion by eliminating barriers to access, improving financial education, and developing financial infrastructure in underserved areas. Digital financial services, including mobile banking, agent banking, and fintech innovations, should be prioritized as mechanisms to expand inclusion without introducing excessive risks. Third, regulatory oversight must be adaptive to account for the evolving relationship between FI and stability. Policymakers should integrate macroprudential regulations that ensure FI initiatives are aligned with broader macroeconomic policies. Strengthening risk management frameworks, particularly for banks expanding into high-risk, financially excluded segments, will be essential. Additionally, governance mechanisms must be reinforced to prevent regulatory capture, ensuring that FI policies remain market-driven and not subject to political distortions. Finally, the regulatory framework governing Ethiopia’s financial sector must evolve to accommodate the growing role of Islamic banking. Given its potential to enhance FI while promoting ethical banking practices, Islamic finance should be supported through a dedicated regulatory framework separate from conventional banking models. Aligning Ethiopia’s Islamic banking regulations with international standards set by institutions such as the Islamic Financial Services Board (IFSB) and the Accounting and Auditing Organization for Islamic Financial Institutions (AAOIFI) will be essential in fostering a stable and inclusive financial ecosystem.

### 5.3 Limitations and future research directions

This study acknowledges the following limitations. First, it does not account for regional disparities within Ethiopia, which may be shaped by the diverse economic and social landscapes across the country’s regions. Future investigations should prioritize a more detailed analysis that incorporates regional policy perspectives, as this would yield richer insights and support the creation of customized interventions aimed at promoting FI both at the regional and national levels. Second, although there were attempts to include digital financial indicators, the lack of transaction data from pertinent authorities hindered the incorporation of these variables into the construction of the index. Consequently, future studies should address this gap. Third, bank stability is measured solely through the Z-score, due to data limitations. Future studies should explore alternative risk-based stability measures. Fourth, while the study employs a two-stage PCA approach, alternative methodologies such as CFA and entropy weighting could further validate the index. Fifth, reliance on commercial bank data may obscure sectoral heterogeneity. Future research should examine how development banks, microfinance institutions, and fintechs navigate the FI-stability trade-off. In particular, the role of digital FI warrants deeper exploration, given its increasing significance in emerging markets. Lastly, a longitudinal approach that tracks the stability effects of FI over an extended period could offer deeper insights into how these dynamics evolve. As regulatory frameworks adapt and financial systems mature, the optimal FI threshold may shift, necessitating ongoing research to refine financial inclusion policies accordingly.

## Ethics and consent

Ethical approval and consent were not required.

## Data Availability

Figshare: Dataset for financial inclusion and stability in Ethiopia case, https://doi.org/10.6084/m9.figshare.27327804.v2 ( Arebo et al., 2024). Data are available under the terms of the Creative Commons Attribution 4.0 International license (CC-BY 4.0).
