# Supplementary material for: Financial inclusion and stability in Ethiopia using bank-level data: A two-step system GMM estimation
Source: F1000Res. 2025 Apr 15;13:1369. Originally published 2024 Nov 15. [Version 2] doi: 10.12688/f1000research.158461.2 (PMC12018872; doi:10.12688/f1000research.158461.2)
Supplement: Peer review history

# Reviewer report: Reviewer response for version 2

**DOI:** 10.5256/f1000research.178988.r378159

**Published:** 2025-04-24

**Authors:** Najabat Ali

**Affiliations:** 1 Soochow University, Suzhou, China

The authors have significantly revised the manuscript; therefore, I recommend it for indexed.

Is the work clearly and accurately presented and does it cite the current literature?

Partly

If applicable, is the statistical analysis and its interpretation appropriate?

Partly

Are all the source data underlying the results available to ensure full reproducibility?

Partly

Is the study design appropriate and is the work technically sound?

Partly

Are the conclusions drawn adequately supported by the results?

Partly

Are sufficient details of methods and analysis provided to allow replication by others?

Partly

Reviewer Expertise:

FDI, Technology Innovation, Green Tarde, Green Innovation, Sustainable Economic Development, Environmental Sustainability

I confirm that I have read this submission and believe that I have an appropriate level of expertise to confirm that it is of an acceptable scientific standard.

# Reviewer report: Reviewer response for version 2

**DOI:** 10.5256/f1000research.178988.r378160

**Published:** 2025-04-23

**Authors:** Hicham Sadok

**Affiliations:** 1 Mohammed V University of Rabat (Morocco), Rabat, Morocco

The authors have taken into account most of the remarks and comments addressed to the first version. They have substantially improved the first version and this new version meets the formal and substantive requirements for acceptance as a scientific article.

Is the work clearly and accurately presented and does it cite the current literature?

Partly

If applicable, is the statistical analysis and its interpretation appropriate?

Yes

Are all the source data underlying the results available to ensure full reproducibility?

Yes

Is the study design appropriate and is the work technically sound?

Yes

Are the conclusions drawn adequately supported by the results?

Partly

Are sufficient details of methods and analysis provided to allow replication by others?

Partly

Reviewer Expertise:

Finance and Economics

I confirm that I have read this submission and believe that I have an appropriate level of expertise to confirm that it is of an acceptable scientific standard.

# Reviewer report: Reviewer response for version 1

**DOI:** 10.5256/f1000research.174050.r362882

**Published:** 2025-02-14

**Authors:** Inès Gharbi

**Affiliations:** 1 University of Sfax, Sfax, Tunisia

Thank you for inviting me to review the current manuscript.

 From my point of view, this is a very interesting topic and a good attempt. I advise the author(s) to continue. Some comments should be taken seriously. I hope that these comments will help to improve the document even more.

 1. The abstract must contain the main contributions.

 2.  I think the introduction should state the novelty of the paper compared to other papers published in the field. Author(s) should be more explicit about how their methodology or results fill identified research gaps to strengthen the justification for publication.

 3.  The paper demonstrates an adequate understanding of the relevant literature in the field. However, I have seen that no hypotheses are mentioned, so it is better to mention the hypotheses and show the reader that you have tested them.

 4. Please give reasons for the choice of methodologies used, why choose the two-stage PCA method rather than the entropy weighting method to construct the Financial Inclusion Index?

 5. At the same time, I think that the conclusion part of the paper should be extended with contributions, limitations, and future research directions.

Is the work clearly and accurately presented and does it cite the current literature?

Yes

If applicable, is the statistical analysis and its interpretation appropriate?

Yes

Are all the source data underlying the results available to ensure full reproducibility?

Yes

Is the study design appropriate and is the work technically sound?

Partly

Are the conclusions drawn adequately supported by the results?

Partly

Are sufficient details of methods and analysis provided to allow replication by others?

Yes

Reviewer Expertise:

Financial technologies, Digitalization, Financial inclusion, renewable energies, and Green finance

I confirm that I have read this submission and believe that I have an appropriate level of expertise to confirm that it is of an acceptable scientific standard, however I have significant reservations, as outlined above.

## References

[1] : Developing a Multidimensional Financial Inclusion Index: A Comparison Based on Income Groups. *Journal of Risk and Financial Management* .2023;16(6) : 10.3390/jrfm16060296 10.3390/jrfm16060296

[2] : Relationship between digital banking and financial inclusion: Evidence from Tunisia. *International Journal of Business Studies* .2022;6(3) : 10.32924/ijbs.v6i3.228 168-177 10.32924/ijbs.v6i3.228

[3] : The Digitalization in the COVID-19 Era. 10.4018/978-1-7998-8674-7.ch015 284-304 10.4018/978-1-7998-8674-7.ch015

# Response

**Published:** 2025-02-14

**Authors:** Mohammed Arebo

**Affiliations:** Regional and Local Development, Addis Ababa University College of Development Studies, Addis Ababa, Addis Ababa, Ethiopia

Dear Ass. Professor Inès Gharbi,

 Thank you very much for dedicating your time and effort to reviewing our work, particularly for your prompt review report. Your insightful comments will undoubtedly enhance the quality of our manuscript. We have taken your comments to heart and will carefully address each of the five main points you raised in the revised version of this article. We look forward to your thoughts on the revisions we have made.

# Response

**Published:** 2025-03-04

**Authors:** Mohammed Arebo

**Affiliations:** Regional and Local Development, Addis Ababa University College of Development Studies, Addis Ababa, Addis Ababa, Ethiopia

Thank you for your detailed and constructive review of our manuscript. Your comments have been instrumental in enhancing the novelty, methodological rigor, and presentation of our work. We have addressed each of your suggestions, as explained below.

 1. **The abstract must contain the main contributions.**

 Response: Thank you for your valuable comment. The abstract has been revised to include the main contributions of the study, particularly the non-linearity test and the analysis of macro-prudential regulation.

 2. **I think the introduction should state the novelty of the paper compared to other papers published in the field. Author(s) should be more explicit about how their methodology or results fill identified research gaps to strengthen the justification for publication.**

 Response: Thank you very much for significant comment. The introduction now explicitly states the novelty of the paper by comparing it with other studies and highlighting how our methodology and results address identified research gaps.

 3. **The paper demonstrates an adequate understanding of the relevant literature in the field. However, I have seen that no hypotheses are mentioned, so it is better to mention the hypotheses and show the reader that you have tested them.**

 Response: Thank you for this significant comment. We updated the literature review with the most recent studies and segmented it to focus on the key variables: (i) financial inclusion and banking stability, (ii) non-linear relationship between financial inclusion and bank stability, (iii) the moderating effects of macro-prudential regulation. This restructuring allows for clearer hypothesis formulation and empirical relevance.

 4. **Please give reasons for the choice of methodologies used, why choose the two-stage PCA method rather than the entropy weighting method to construct the Financial Inclusion Index?**

 Response: Thank you this valuable comment. We have provided a rationale for choosing the two-stage PCA method over alternatives, such as the entropy weighting and CFA method, for constructing the financial inclusion index.

 5. **At the same time, I think that the conclusion part of the paper should be extended with contributions, limitations, and future research directions.**

 Response: Thank you for your significant comment. The conclusion has been expanded considering contribution. Policy implications, and limitations and future research directions presented in separate section.

# Reviewer report: Reviewer response for version 1

**DOI:** 10.5256/f1000research.174050.r362883

**Published:** 2025-02-11

**Authors:** Hicham Sadok

**Affiliations:** 1 Mohammed V University of Rabat (Morocco), Rabat, Morocco

The article examines the impact of financial inclusion on banking stability in the Ethiopian context. It addresses an important issue that deserves analysis and investigation. However, some elements deserve improvement:

 1-To clarify in the introduction the two key variables of the study, namely what is meant by financial inclusion and banking stability: these two concepts are too broad and must be defined precisely.

 2- From the conceptualization of the key variables, it would be more structuring in the literature review to focus on the work related to these two concepts in order to be able to ultimately deduce ideas or hypotheses to study in the empirical part: a segmented literature review like what has been done does not allow us to know what the usefulness of this step is for the future!!

 3-It would be useful in the empirical part to detail a little more on the stylized facts of the banks studied.

 4- At the level of dependent variables, namely, financial stability, the Z score is not sufficiently significant for stability, it rather designates profitability. However, if the latter is important for stability, it is not sufficient. Why not consider the ratios of the base as a criterion of stability instead of the Z ratio?

 Good luck

Is the work clearly and accurately presented and does it cite the current literature?

Partly

If applicable, is the statistical analysis and its interpretation appropriate?

Yes

Are all the source data underlying the results available to ensure full reproducibility?

Yes

Is the study design appropriate and is the work technically sound?

Yes

Are the conclusions drawn adequately supported by the results?

Partly

Are sufficient details of methods and analysis provided to allow replication by others?

Partly

Reviewer Expertise:

Finance and Economics

I confirm that I have read this submission and believe that I have an appropriate level of expertise to confirm that it is of an acceptable scientific standard, however I have significant reservations, as outlined above.

## References

[1] : The Contribution of AI-Based Analysis and Rating Models to Financial Inclusion.2023; 10.1201/9781003125204-2 11-25 10.1201/9781003125204-2

[2] : How can inclusive growth be enabled from financial technology. *International Journal of Business Performance Management* .2021;22(2/3) : 10.1504/IJBPM.2021.116410 10.1504/IJBPM.2021.116410

# Response

**Published:** 2025-02-12

**Authors:** Mohammed Arebo

**Affiliations:** Regional and Local Development, Addis Ababa University College of Development Studies, Addis Ababa, Addis Ababa, Ethiopia

Dear Professor Hicham Sadok,

 We sincerely thank you for the time and effort you have dedicated to reviewing our work, particularly for your prompt review report. Your insightful comments will undoubtedly enhance the quality of our manuscript. We have taken your four key comments into consideration in the revised version of this article.

# Response

**Published:** 2025-03-04

**Authors:** Mohammed Arebo

**Affiliations:** Regional and Local Development, Addis Ababa University College of Development Studies, Addis Ababa, Addis Ababa, Ethiopia

We sincerely appreciate the time and effort you have taken to review our manuscript. Your thoughtful comments have provided valuable guidance, enabling us to strengthen the theoretical, empirical and methodological aspects of the study. Below, we outline the changes made in response to your feedback.

 1.    To clarify in the introduction, the two key variables of the study, namely what is meant by financial inclusion and banking stability: these two concepts are too broad and must be defined precisely.

 Response: Thank you for your valuable comment.  The introduction has been revised to provide precise definitions of financial inclusion and banking stability.

 2.    From the conceptualization of the key variables, it would be more structuring in the literature review to focus on the work related to these two concepts in order to be able to ultimately deduce ideas or hypotheses to study in the empirical part: a segmented literature review like what has been done does not allow us to know what the usefulness of this step is for the future!!

 Response: Thank you for this significant comment. We updated the literature review with the most recent studies and segmented it to focus on the key variables: (i) financial inclusion and banking stability, (ii) non-linear relationship between financial inclusion and bank stability, (iii) the moderating effects of macro-prudential regulation. This restructuring allows for clearer hypothesis formulation and empirical relevance.

 3.    It would be useful in the empirical part to detail a little more on the stylized facts of the banks studied.

 Response: Thank you very much your feedback. Additional details on the stylized facts of the banks studied have been included in the empirical section.

 4.    At the level of dependent variables, namely, financial stability, the Z score is not sufficiently significant for stability, it rather designates profitability. However, if the latter is important for stability, it is not sufficient. Why not consider the ratios of the base as a criterion of stability instead of the Z ratio?

 Response: Thank you for your insightful comment. While the Z-score may overemphasize profitability, it was used due to the unavailability of risk-related data. We have acknowledged this limitation in the revised manuscript, stating: “Bank stability is measured solely through the Z-score due to data limitations. Future studies should explore alternative risk-based stability measures.”

# Reviewer report: Reviewer response for version 1

**DOI:** 10.5256/f1000research.174050.r354020

**Published:** 2025-01-22

**Authors:** Najabat Ali

**Affiliations:** 1 Soochow University, Suzhou, China

Thanks for inviting me to review the current manuscript. The manuscript has some merits, but its current quality does not meet the indexing standard. Therefore, before indexing, the manuscript needs the following significant modifications:

 1. The authors must add a specific policy recommendation in the abstract section.

 2. The study's novelty should be highlighted by comparing the current study with previous studies in the field.

 3. The theoretical framework should be added.

 4. The literature review section should be enriched with the most recent studies.

 5. In the methodology section, justify the methods used in your study by comparing them with the existing field methods.

 6. The graphical presentation of the methodology will be easier for the authors to understand.

 7. In the results and discussion section, compare your results with the existing field studies.

 8 The conclusion, policy implications, and limitations should be written under separate headings.

 9. An English language check is required to avoid grammatical and spelling errors.

Is the work clearly and accurately presented and does it cite the current literature?

Partly

If applicable, is the statistical analysis and its interpretation appropriate?

Partly

Are all the source data underlying the results available to ensure full reproducibility?

Partly

Is the study design appropriate and is the work technically sound?

Partly

Are the conclusions drawn adequately supported by the results?

Partly

Are sufficient details of methods and analysis provided to allow replication by others?

Partly

Reviewer Expertise:

FDI, Technology Innovation, Green Tarde, Green Innovation, Sustainable Economic Development, Environmental Sustainability

I confirm that I have read this submission and believe that I have an appropriate level of expertise to confirm that it is of an acceptable scientific standard, however I have significant reservations, as outlined above.

## References

[1] : Determinants of Fintech Adoption in Banking: The Role of Technological Literacy: Evidence from Pakistan. *International Journal of Technological Learning, Innovation and Development* .2025;16(3) : 10.1504/IJTLID.2025.10066896 10.1504/IJTLID.2025.10066896

[2] : The impact of digital financial development on corporate leverage ratio: The case of a-share listed non-financial enterprises in China's Shanghai and Shenzhen stock exchanges. *PLoS One* .2024;19(8) : 10.1371/journal.pone.0302978 e0302978 10.1371/journal.pone.0302978 39133746 PMC11318853

# Response

**Published:** 2025-01-24

**Authors:** Mohammed Arebo

**Affiliations:** Regional and Local Development, Addis Ababa University College of Development Studies, Addis Ababa, Addis Ababa, Ethiopia

Dear Professor Najabat Ali,

 We sincerely thank you for the time and effort you have dedicated to reviewing our work, particularly for your prompt review report. Your insightful comments will undoubtedly enhance the quality of our manuscript. We eagerly anticipate additional feedback and suggestions from the other reviewers, and upon receipt, we will take your nine comments into account in the revised version of this article.

 Regards

 Mohammed Jatoro Arebo

 Main and corresponding author

# Response

**Published:** 2025-03-04

**Authors:** Mohammed Arebo

**Affiliations:** Regional and Local Development, Addis Ababa University College of Development Studies, Addis Ababa, Addis Ababa, Ethiopia

Thank you for your thorough review and constructive feedback on our manuscript. Your insightful comments have significantly helped us improve the clarity, rigor, and overall quality of the study. We have carefully addressed each of your suggestions, as detailed below.

 1.    The authors must add a specific policy recommendation in the abstract section.

 Response: We appreciate your valuable comment. We added a specific policy recommendation to the abstract section.

 2.    The study's novelty should be highlighted by comparing the current study with previous studies in the field.

 Response: Thank you for your important comment. We highlighted the novelty of the study by comparing it with previous research and explicitly stating how our methodology and results address identified research gaps.

 3.    The theoretical framework should be added.

 Response: Thank you for your meticulous comment. We enriched the theoretical framework to provide a stronger foundation for the study.

 4.    The literature review section should be enriched with the most recent studies.

 Response: Thank you for this significant comment. We updated the literature review with the most recent studies and segmented it to focus on the key variables: (i) financial inclusion and banking stability, (ii) non-linear relationship between financial inclusion and bank stability, (iii) the moderating effects of macro-prudential regulation. This restructuring allows for clearer hypothesis formulation and empirical relevance.

 5.    In the methodology section, justify the methods used in your study by comparing them with the existing field methods.

 Response: Thank you for your valuable comment. We have justified the methods used in the study by comparing them with existing approaches (specifically with FE and RE) in the field. The methodology section, previously included in the results, has been repositioned to the Model Specification subsection under the Methods section. Additionally, we have introduced the non-linearity test and Lind and Mehlum’s (2010) U-test in this section to enhance methodological rigor. Lastly, we have provided a rationale for choosing the two-stage PCA method over alternatives, such as the entropy weighting and CFA method, for constructing the financial inclusion index.

 6.    The graphical presentation of the methodology will be easier for the authors to understand.

 Response: Thank you for your comment. A graphical representation of the methodology has been added to improve clarity and accessibility.

 7.    In the results and discussion section, compare your results with the existing field studies.

 Response: Thank you very much for this significant comment. The results and discussion section now includes comparisons with existing studies to contextualize our findings.

 8.    The conclusion, policy implications, and limitations should be written under separate headings.

 Response: Thank you for your significant comment. In line with the journal guidelines and other reviewer comments, the conclusion restructured into separate sections for policy implications, limitations, and future research directions, with the study’s contributions explicitly highlighted within the conclusion.

 9.    An English language check is required to avoid grammatical and spelling errors.

 Response: Thank you for your significant comment. A thorough English language check has been conducted to eliminate grammatical and spelling errors.
